# The Evolutionary Origination and Diversification of a Dimorphic Gene Regulatory Network through Parallel Innovations in *cis* and *trans*

**DOI:** 10.1371/journal.pgen.1005136

**Published:** 2015-04-02

**Authors:** Eric M. Camino, John C. Butts, Alison Ordway, Jordan E. Vellky, Mark Rebeiz, Thomas M. Williams

**Affiliations:** 1 Department of Biology, University of Dayton, Dayton, Ohio, United States of America; 2 Department of Biological Sciences, University of Pittsburgh, Pittsburgh, Pennsylvania, United States of America; 3 Center for Tissue Regeneration and Engineering at Dayton, University of Dayton, Dayton, Ohio, United States of America; University of California Berkeley, United States of America

## Abstract

The origination and diversification of morphological characteristics represents a key problem in understanding the evolution of development. Morphological traits result from gene regulatory networks (GRNs) that form a web of transcription factors, which regulate multiple *cis*-regulatory element (CRE) sequences to control the coordinated expression of differentiation genes. The formation and modification of GRNs must ultimately be understood at the level of individual regulatory linkages (i.e., transcription factor binding sites within CREs) that constitute the network. Here, we investigate how elements within a network originated and diversified to generate a broad range of abdominal pigmentation phenotypes among *Sophophora* fruit flies. Our data indicates that the coordinated expression of two melanin synthesis enzymes, Yellow and Tan, recently evolved through novel CRE activities that respond to the spatial patterning inputs of Hox proteins and the sex-specific input of Bric-à-brac transcription factors. Once established, it seems that these newly evolved activities were repeatedly modified by evolutionary changes in the network’s *trans*-regulators to generate large-scale changes in pigment pattern. By elucidating how *yellow* and *tan* are connected to the web of abdominal *trans*-regulators, we discovered that the *yellow* and *tan* abdominal CREs are composed of distinct regulatory inputs that exhibit contrasting responses to the same Hox proteins and Hox cofactors. These results provide an example in which CRE origination underlies a recently evolved novel trait, and highlights how coordinated expression patterns can evolve in parallel through the generation of unique regulatory linkages.

## Author Summary

The genomic content of regulatory genes such as transcription factors is surprisingly conserved between diverse animal species, raising the paradox of how new traits emerge, and are subsequently modified and lost. In this study we make a connection between the developmental basis for the formation of a fruit fly trait and the evolutionary basis for that trait’s origin, diversification, and loss. We show how the origin of a novel pigmentation trait is associated with the evolution of two regulatory sequences that control the co-expression of two key pigmentation genes. These sequences interact in unique ways with evolutionarily conserved Hox transcription factors to drive gene co-expression. Once these unique connections evolved, the alteration of this trait appears to have proceeded through changes to regulatory genes rather than regulatory sequences of the pigmentation genes. Thus, our findings support a scenario where regulatory sequence evolution provided new functions to old transcription factors, how co-expression can emerge from different utilizations of the same transcription factors, and that trait diversity was surprisingly shaped by changes in some manner to the deeply conserved regulatory genes.

## Introduction

The complexity of developmental processes often hinders our ability to trace their evolutionary history. Genetic programs of development are structured into convoluted networks of genes, interconnected at the level of transcriptional regulation [1]. Each network connection, or regulatory linkage, is formed through interactions between a transcription factor protein and binding site sequences within a *cis*-regulatory element (CRE). The collection of regulatory linkages possessed by a CRE encodes the pattern of gene expression driven by the CRE. Networks culminate in the regulation of differentiation genes whose encoded products generate cell type-specific phenotypes. Hence, to understand how a developmental program originated or was diversified, one must trace how individual connections were formed between transcription factors and the CREs of the network.

CRE evolution is suspected to be a prime mode of trait evolution [2–4], and in recent years several case studies have described CREs that have been modified to generate phenotypic consequences [5–19]. However, our current understanding of network evolution is hampered by the general difficulty of resolving the direct regulatory linkages within CREs and how CRE mutations alter specific linkages [20]. For example, when genes are coordinately expressed, how similar are the encoded regulatory linkages within their CREs? What factors preside over the tendency of a network to evolve at upper level regulators or the terminal differentiation genes? The answers to these questions require studies of well-defined networks that govern morphologies that have diversified during recent evolutionary history.

**Fig 1:**
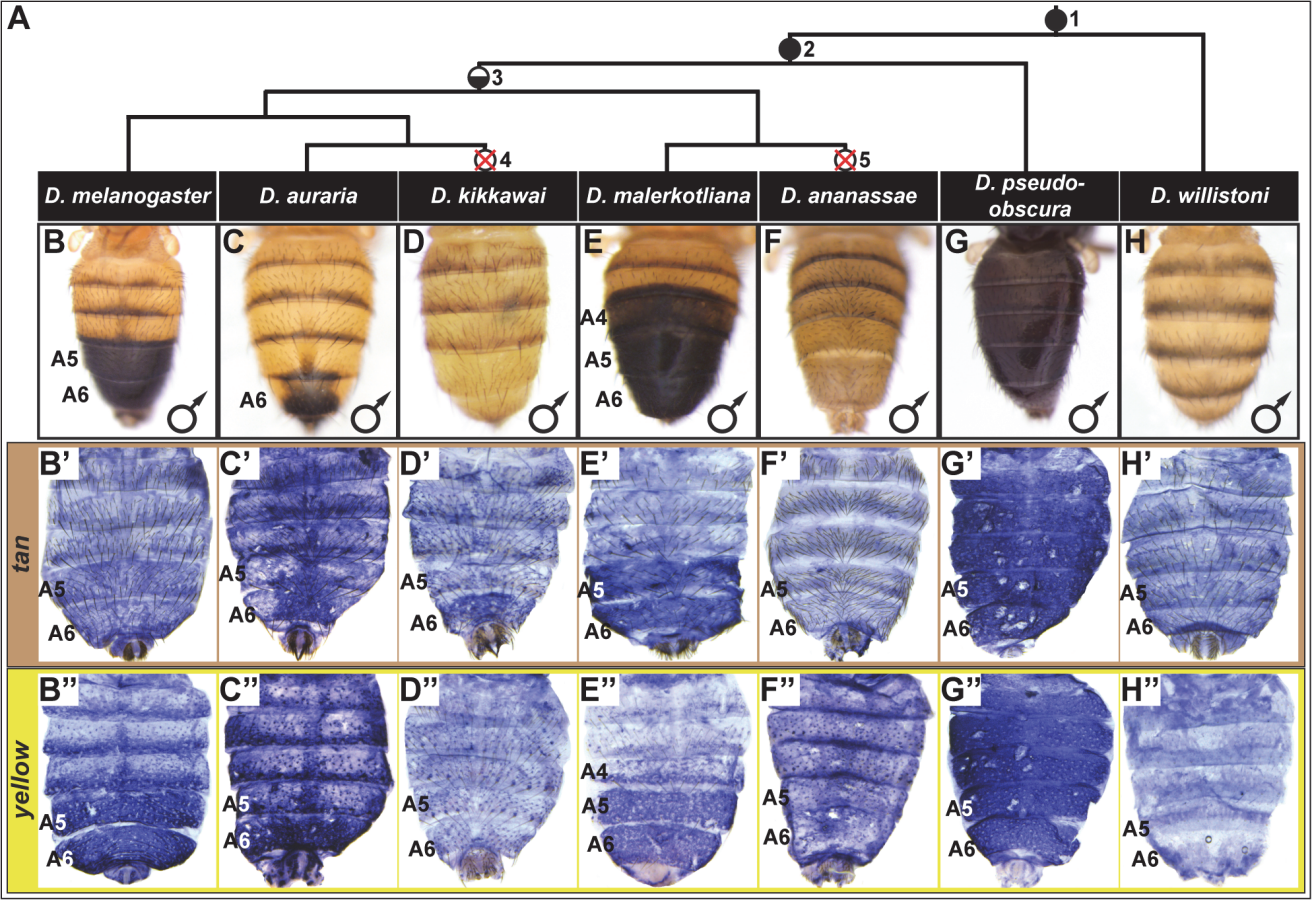
Correlation between pigmentation and the gene expression of *tan* and *yellow* in the *Sophophora* subgenus. (A) Phylogenetic relationship between extant *Sophophora* species. (B-H, B’-H’, and B”-H’) Each image shown is from a male abdomen. Whole-mount images of dorsal fruit fly abdomens from species representing diverse *Sophophora* lineages. *D*. *melanogaster* bears a derived pigmentation pattern on the A5 and A6 tergites, which arose after it diverged from its most recent common ancestor (MRCA) shared with the monomorphically pigmented *D*. *willistoni* (node in phylogeny marked “1”) and perhaps after diverging from the MRCA shared with *D*. *pseudoobscura* (node “2”). Node 3 represents the MRCA of the *melanogaster* species group that includes the Oriental lineage (*D*. *melanogaster*), *montium* subgroup (includes *D*. *auraria* and *D*. *kikkawai*), and *ananassae* subgroup (includes *D*. *malerkotliana* and *D*. *ananassae*). This MRCA is suspected to have possessed male-specific tergite pigmentation that is indicated by the hemi-filled in circle. Since its origin, the number of pigmented male tergites has expanded (*D*. *malerkotliana*), retracted (*D*. *auraria*) and was independently lost (*D*. *kikkawai* and *D*. *ananassae*; nodes 4 and 5 that are indicated by the circles with a superimposed X). (B’-H’) Abdominal *tan* mRNA expression shown by *in situ* hybridization at a developmental stage equivalent to 85–95 hours after puparium formation (APF) for *D*. *melanogaster* pupae. (B”-H”) Abdominal *yellow* mRNA expression shown by *in situ* hybridization at a developmental stage equivalent to 75–85 hours APF for *D*. *melanogaster* pupae. Species are identified by labels at the top of each column.

The diverse abdominal pigmentation patterns of fruit fly species represent an optimal model to study the evolution of morphological characteristics [21]. The model organism *Drosophila* (*D*.) *melanogaster* belongs to the fruit fly subgenus *Sophophora* [22], which contains species with a wide diversity of abdominal pigmentation patterns (Fig 1). A central theme that typifies *D*. *melanogaster* and its close relatives (the *melanogaster* species group) is dimorphism, in which darkly pigmented males differ substantially from the generally unpigmented females. An ancestral character reconstruction analysis supports (84% posterior probability) an evolutionary scenario in which the most recent common ancestor of the *melanogaster* species group possessed a male-specific pattern of abdomen pigmentation (Fig 1, node 3)[23]. Unlike the *melanogaster* species group, monomorphic pigmentation is predominant among extant species in the more distantly-related *obscura* (e.g. *D*. *pseudoobscura*) and *willistoni* (e.g. *D*. *willistoni*) species groups, supporting the scenario that the most recent common ancestor of the *melanogaster*, *obscura*, and *willistoni* species groups had a monomorphic pattern of pigmentation (Fig 1, node 1) [23]. However, it remains uncertain whether the most recent common ancestor of the *melanogaster* and *obscura* group species (Fig 1, node 2) had dimorphic or monomorphic abdomen pigmentation. Once dimorphic pigmentation arose, the patterns diversified, expanding and contracting along the body axis in Oriental (e.g. *D*. *melanogaster*), *montium* (e.g. *D*. *auraria*), and *ananassae* (e.g. *D*. *malerkotliana*) clades (Fig 1) [23–25]. Within the *melanogaster* species group, several instances of reversion to the monomorphic state occurred, as exemplified by *D*. *kikkawai* (*montium* subgroup) and *D*. *ananassae* (*ananassae* subgroup) (Fig 1, nodes 4 and 5). Collectively, *Sophophora* tergite pigmentation provides an optimal model to investigate trait evolution, especially considering the extensively characterized network and CREs governing the development of pigmentation in *D*. *melanogaster*.

Within the abdomen of *D*. *melanogaster*, coloring of the dorsal cuticular plates (tergites) requires the co-expression of the genes *tan* and *yellow* in the underlying epidermal cells (Fig 1B’ and 1B”) [26]. Robust expression of *tan* and *yellow* in the male A5 and A6 segments are respectively regulated by CREs known as the *tan* male specific element (t_MSE) [26] and the *yellow* body element (yBE) [23,27]. The Hox protein Abd-B, expressed in the pigmented A5 and A6 segments, is a direct activator of the yBE [23], and represents a likely regulator of *tan*. However, little else is known about the regulatory linkages encoding the spatial and temporal activities for these CREs.

In this study we investigated the evolutionary histories and regulatory encodings of the yBE and t_MSE that coordinate expression of the Tan and Yellow pigmentation enzymes. Our results indicate that these CREs originated at different time points in the lineage leading to the common ancestor of the *melanogaster* species group. Our data supports a scenario where expansions, contractions, and losses of male-specific pigmentation evolved through a preponderance of *trans*-regulatory changes to the abdominal pigmentation gene network. In dissecting *trans*-regulatory inputs to the yBE and t_MSE, we discovered that these two CREs respond differently to alterations of the *trans*-landscape, notwithstanding their superficially similar patterns of expression. Lastly, our results indicate that these differences in responsiveness may be due to unique binding site architectures at these CREs, including a novel mechanism for the regulation of the t_MSE by the Hox protein Abd-A.

## Results

### The evolution of *tan* and *yellow* expression patterns

In *D*. *melanogaster*, *tan* and *yellow* are required for the pigmentation that develops on the male A5 and A6 segment tergites [26,28,29], and these genes’ expression patterns in the dorsal epidermis underlying the tergites closely matches the pattern of pigmentation (compare Fig 1B’ and 1B” to Fig 1B). For *D*. *melanogaster*, this is both the first report of *tan* expression and the first report of the RNA expression pattern for *yellow*. It has been shown that a similar expansion in *tan* and *yellow* expression occurs in *D*. *prostipennis*, a species that displays pigmentation extending into the male A4 tergite [30]. Additionally, the loss of pigmentation in *D*. *santomea* was accompanied by the joint loss of *yellow* and *tan* expression [23,26]. Thus, we anticipated that the origin, diversification, and loss of male pigmentation among the *Sophophora* subgenus will have been driven in part by physically corresponding changes in *tan* and *yellow* expression.

For *D*. *auraria*, we found that *tan* and *yellow* expression is limited to the dorsal epidermis of the male A6 segment (Fig 1C’ and 1C”), the only segment in this species that manifests male-specific pigmentation. While *yellow* expression occurs throughout the A6 segment, the hemispherical pattern of *tan* expression more-closely matches that of the pigmentation pattern, suggesting that *tan* plays an important role in the spatial-limitation of this male pattern element. For *D*. *malerkotliana*, we found that the expression of *yellow* (Fig 1E”) extends into the A4 segment that exhibits expanded pigmentation (Fig 1E). However, *tan* expression remained restricted to the A5 and A6 segments (Fig 1E’), suggesting that the evolved pattern of *yellow* expression plays a role in this derived phenotype, and the absence of *tan* expression may explain why the A4 tergite has less intense pigmentation than that present on the A5 and A6 tergites.

Pigmentation has been secondarily lost from *D*. *kikkawai* and *D*. *ananassae* males, which may have resulted from the loss of expression of *tan* and *yellow*. While *yellow* expression was found to be absent from the abdomen of *D*. *kikkawai* (Fig 1D”), *tan* expression was observed in the A6 segment in a pattern similar to that for *D*. *auraria* (compare Fig 1C’ and 1D’). This implies that the loss of male-specific pigmentation for this species included the loss of *yellow* expression, but did not require the inactivation of *tan* expression. For *D*. *ananassae*, neither *tan* nor *yellow* were found to be expressed in the male abdominal epidermis (Fig 1F’ and 1F”). It remains possible that these cases of pigmentation loss were initially due to a mechanism that had no bearing on *yellow* and *tan* expression. At a minimum, our results suggest that pigmentation loss at some point was accompanied by changes that eliminated these genes expression from the abdominal epidermis. This outcome was previously shown to have occurred in *D*. *santomea* [23,26], a third *Sophophora* species for which male tergite pigmentation was independently lost [31].

The aforementioned expression patterns support a role for changes in *tan* and *yellow* expression in the diversification and secondary loses of male pigmentation within the *melanogaster* species group. To date though, the expression of these pigmentation genes had not been investigated in species from the *obscura* and *willistoni* species groups in which most known extant species possess sexually monomorphic patterns of tergite pigmentation. For *D*. *pseudoobscura* of the *obscura* species group, we found that *tan* and *yellow* are both expressed throughout the male abdominal epidermis (Fig 1G’ and 1G”), a pattern that corresponds with this species’ dark coloration (Fig 1G). For *D*. *willistoni*, of the *willistoni* species group, we found that neither *yellow* nor *tan* are expressed at appreciable levels in the abdominal epidermis during the stages when tergite pigmentation is being specified (Fig 1H’ and 1H”). This suggests that *D*. *willistoni* lacks pigmentation in part due to the absence of these genes’ expression, and this monomorphic absence may reflect the ancestral state from which male-specific pigmentation evolved, as suggested elsewhere [6,16,23,32,33].

Collectively, our expression analyses support the hypothesis that the origin, diversification, and loss of male-specific pigmentation involved numerous alterations to the expression of *tan* and *yellow*. The mutational and mechanistic basis for CRE evolution remains poorly understood, especially in cases for genes whose expression patterns physically correspond. Thus, we next sought to determine the CRE basis for these evolved gene expression patterns and phenotypic differences.

### The evolutionary origin of CREs controlling *tan* and *yellow* expression

**Fig 2:**
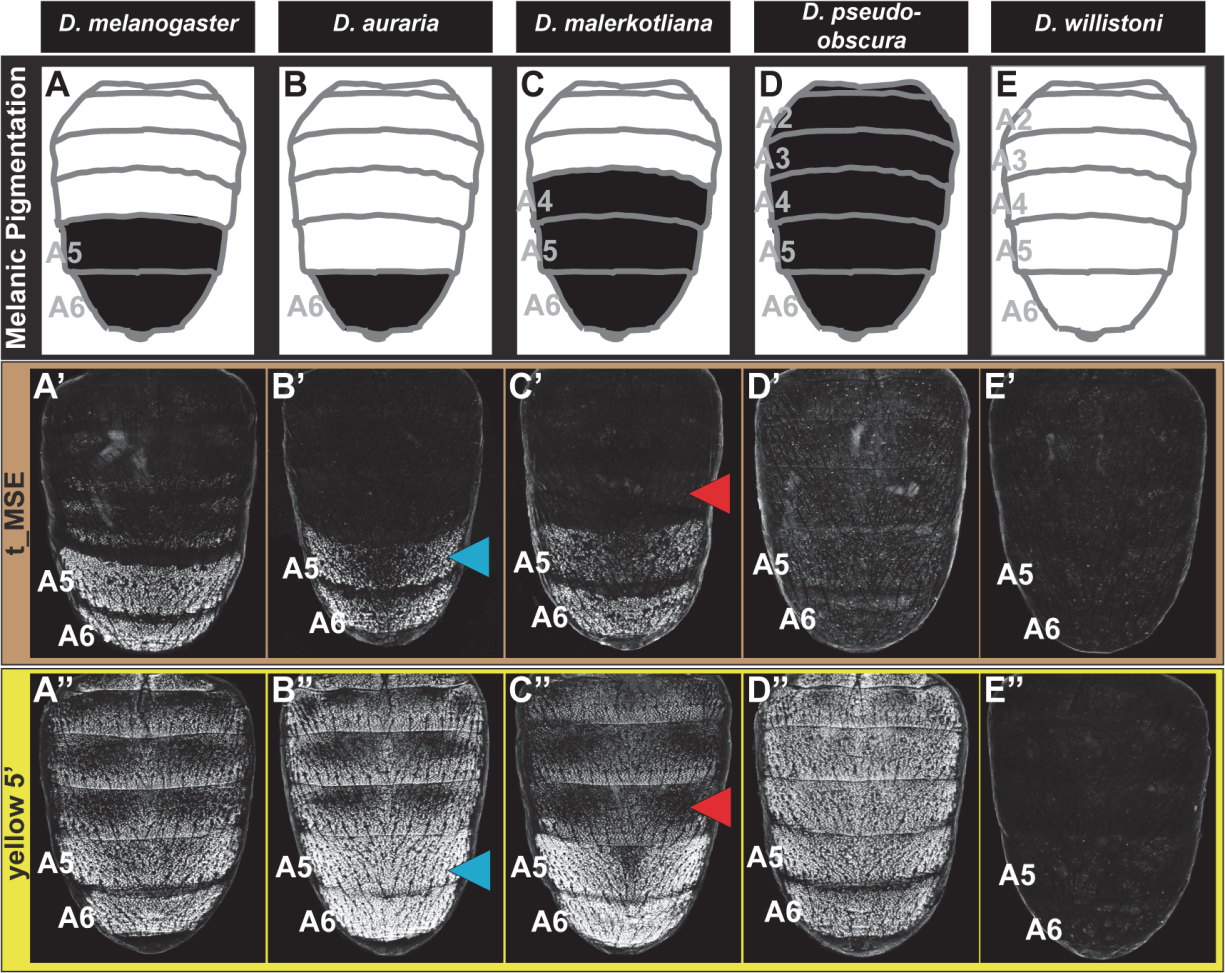
Tracing the ancestry and evolution of CREs that drive male-specific *tan* and *yellow* expression. (A-E) A schematic representation of the male abdomens for several *Sophophora* species with diverse patterns of pigmentation. (A’-E’) EGFP-reporter transgene expression driven by sequences orthologous to the *D*. *melanogaster* t_MSE in transgenic male pupae at ~95 hours after puparium formation. (A’-C’) Sequences from species with male-specific tergite pigmentation drive robust male-specific reporter expression in the A5 and A6 segments, (D’ and E’) whereas the sequences from more distantly related species with monomorphic tergite pigmentation have little-to-no abdominal regulatory activity. (A”-E”) EGFP-reporter transgene expression driven by sequences orthologous to the *D*. *melanogaster yellow* wing/body element, here referred to as *yellow* 5’, in transgenic male pupae at ~85 hours after puparium formation. (A”-C”) Sequences from species with male-specific tergite pigmentation drive robust male-specific reporter expression in the A5 and A6 segments. (D”) The *D*. *pseudoobscura* sequence drives pan-abdomen reporter expression. (E”) The *D*. *willistoni* sequence lacks abdominal regulatory activity. Blue arrowheads indicate *D*. *auraria* reporter expression in transgenic *D*. *melanogaster* that extends one segment anterior to the endogenous domain of pigmentation. Red arrowheads indicate the absence of *D*. *malerkotliana* reporter expression in the A4 segment of transgenic *D*. *melanogaster*, a segment that is endogenously pigmented.

The recent evolution of sexually dimorphic pigmentation within the subgenus *Sophophora* provides a model trait whose origination can be resolved to the level of CREs activating pertinent genes within a network. We sought to elucidate the evolutionary histories of the yBE and t_MSE that respectively control the coordinated expression of the *yellow* and *tan* genes. The sequences orthologous to these *tan* and *yellow* abdominal CREs were isolated from species with the derived male-specific tergite pigmentation, as well as species from more distantly-related lineages and that possess monomorphic patterns of tergite pigmentation. For these sequences, we directly compared their regulatory activities in *in vivo* reporter transgene assays in *D*. *melanogaster* (Fig 2).

The region orthologous to the t_MSE (S1A Fig) was isolated from the dimorphically pigmented species *D*. *auraria* (*montium* subgroup), and *D*. *malerkotliana* (*ananassae* subgroup), and tested for abdominal activity in transgenic *D*. *melanogaster* pupae. In each case, robust reporter expression was observed in the male A5 and A6 abdomen segments (Fig 2B’ and 2C’). Likewise, orthologous sequences containing the yBE CREs derived from these same species each drove a male-specific pattern of reporter gene expression (Fig 2B” and 2C”). These similarities in regulatory activities to the *D*. *melanogaster* CREs support a scenario in which the common ancestor of the *melanogaster* species group (Fig 1, node 3) possessed male-specific *tan* and *yellow* gene expression driven respectively by an ancestral t_MSE and yBE.

To determine the timing and mechanism by which the novel trait of sexually dimorphic pigmentation arose, we isolated and tested orthologous sequences from the genomes of *D*. *pseudoobscura* and *D*. *willistoni*, two species from inferred ancestrally monomorphic lineages. As the t_MSE lies in between two upstream genes (*CG1537* and *Gr8a*, S1A Fig), we confirmed their syntenic organization in these species (S2 Fig), suggesting a conserved gene order in the common ancestor of these disparate *Sophophora* lineages. However, the *D*. *pseudoobscura* and *D*. *willistoni* intergenic sequences between *CG1537* and *Gr8a* had little-to-no t_MSE-like CRE activity (Fig 2D’ and 2E’). The absence of CRE activity parallels our observations of *tan* expression for *D*. *willistoni* (Fig 1H’), but contrasts with the monomorphic pattern of expression observed for the monomorphically pigmented *D*. *pseudoobscura* (Fig 1G’). These results are consistent with a scenario where the t_MSE originated to generate dimorphic expression in the lineage leading to the *melanogaster* species group after it diverged from the *obscura* group lineage (Fig 1, node 3). Moreover, the monomorphic expression of *tan* for *D*. *pseudoobscura* seemingly would be driven by another regulatory sequence or sequences.

The sequence 5’ of the *D*. *pseudoobscura yellow* gene possessed abdominal CRE activity that was enhanced in males compared to females (S3 Fig), though the domain of activity spanned all abdominal segments (Fig 2D”). Previously, a pan-abdomen CRE activity was observed for *D*. *pseudoobscura* [34] and for the orthologous gene region from *D*. *subobscura*, another *obscura* group species [23]. The results here show that the *D*. *pseudoobscura* sequence has abdominal regulatory activity that is enhanced in males when assayed in the *D*. *melanogaster trans*-regulatory environment. This suggests that a CRE with spatial and sex-specific inputs was present in the *yellow* gene of the most recent common ancestor of the *obscura* and *melanogaster* species groups (Fig 1, node 2).

To determine whether the yBE has an even deeper *Sophophora* ancestry, we inspected the regulatory capability of sequences *5’* of the *D*. *willistoni yellow* gene (S4 Fig). Since little-to-no sequence conservation is detectable in comparisons of *D*. *willistoni yellow* 5’ sequence to that of *D*. *pseudoobscura* and *D*. *melanogaster*, we evaluated the regulatory activity of two partially overlapping (1.2 kilobase, or kb, overlap) sequences that collectively span the first 5.1 kb of sequence 5’ of *yellow* exon 1 (S4A Fig). The proximal 3 kb to *yellow* exon 1 (called y wil 5’ 2) lacked abdominal regulatory activity (S4C Fig), whereas the more distal (y wil 5’ 1) sequence had CRE activity limited to monomorphic stripes at the posterior edges of each abdominal segment (S4B Fig) and throughout the pupal wing (S4B’ Fig). These stripe and wing activities are characteristic of the *D*. *melanogaster* wing element CRE that is similarly positioned more distal to the 1^st^ exon of *yellow* than the yBE [27] and corresponds with the location of this species tergite pigmentation (Fig 1H). Thus, the *D*. *willistoni yellow* locus possesses an orthologous wing element, but lacks a CRE with activity characteristic of the yBE. This *D*. *willistoni* CRE architecture can also be inferred from a previous study that looked at 5.9 kb of 5’ sequence in a single reporter transgene [34]. These results support an evolutionary scenario where the most recent common ancestor of monomorphic and dimorphic *Sophophora* lineages (Fig 1, node 1) lacked an orthologous body element. Moreover, the evolution of sexually dimorphic pigmentation was accompanied by the origination of novel CRE activities of *yellow* and *tan* that integrate spatial and sex-specific regulatory inputs, and the t_MSE appears to be of more recent origin than the yBE.

### Expansion and contraction of pigment patterns through *trans* evolution

Following the origin of male-specific tergite pigmentation, the number of pigmented tergites expanded and contracted to range from the single A6 segment of *D*. *auraria*, to the A5 and A6 segments of *D*. *melanogaster*, and the A4-A6 segments seen for *D*. *malerkotliana* (Fig 1). These phenotypic changes correspond with expansions and retractions in the expression of *tan* (Fig 1B’, 1C’, and 1E’) and *yellow* (Fig 1B”, 1C”, and 1E”) along the anterior-posterior axis. *A priori*, such changes in spatial expression could originate from sequence changes in the t_MSE and yBE (hereafter referred to as “*cis*-evolution”) or through changes in an upstream regulatory gene or genes (referred to hereafter as “*trans*-evolution”). We found that the t_MSE and *yellow* 5’ regulatory sequences from *D*. *auraria* each drove reporter gene expression in the male A5 and A6 segments of transgenic *D*. *melanogaster* (Fig 2B’ and 2B”). This domain of activity matches the output driven by the *D*. *melanogaster* CREs, yet extends one segment anterior relative to their endogenous expression in *D*. *auraria* (compare Fig 2B’ and 2B” to Fig 1C’ and 1C”). Similarly, the t_MSE and *yellow* 5’ sequences for *D*. *malerkotliana* each drove reporter expression in the A5 and A6 segments of transgenic *D*. *melanogaster* (Fig 2C’ and 2C”), a domain that is shifted posterior by one segment compared to the pigmentation phenotype and that for the endogenous pattern of *yellow* expression in *D*. *malerkotliana* (Fig 1E”). The up-regulation of *yellow* in the *D*. *malerkotliana* A4 segment is modest relative to the A5 and A6 segments, matching the lighter phenotype of this segment (Fig 1E). However, the *D*. *malerkotliana yellow* 5’ sequence is functionally-indistinguishable from the *D*. *melanogaster* CRE in the A4 segment. This similarity could be explained by “*cis*” evolution elsewhere in the *yellow* locus, such as the wing element, promoter, or intron. However, the *yellow* 5’ sequences evaluated here included the wing element and the putative *D*. *malerkotliana* promoter region. Thus, such a “*cis*-elsewhere” scenario would have to be due to an evolved intronic or more distally-located CRE. We favor the interpretation that the *D*. *malerkotliana* expression phenotype arose by evolution in *trans*, and more broadly that diversification in male tergite pigmentation evolved in large part from changes in *trans* to *tan* and *yellow*. In the future, the reciprocal test of the orthologous sequences as reporter transgenes in *D*. *malerkotliana* could provide more definite evidence for either the *cis*-elsewhere in *yellow* or *trans*-evolution scenarios.

### Pigmentation loss through a mosaic of *cis*- and *trans*-evolution

**Fig 3:**
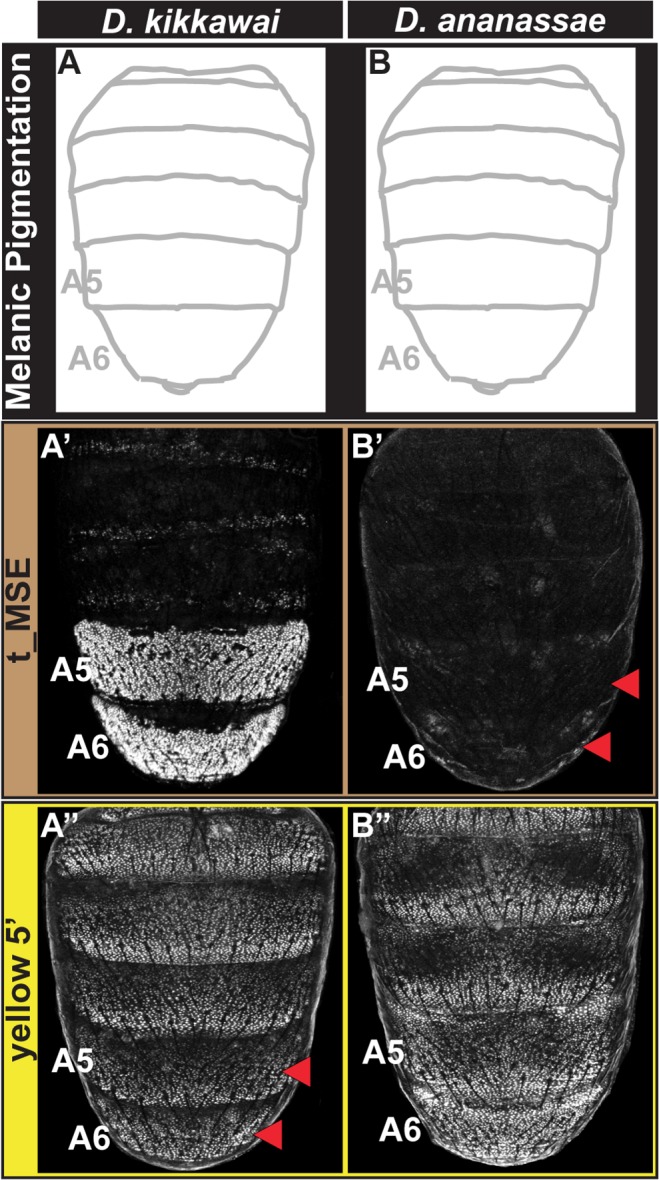
Tracing the CRE bases for losses in male tergite pigmentation. (A and B) A schematic representation of the male abdomens for two species with derived losses in male pigmentation. (A’ and B’) EGFP-reporter transgene expression driven by sequences orthologous to the *D*. *melanogaster* t_MSE in transgenic male pupae at ~95 hours after puparium formation. (A’) The *D*. *kikkawai* sequence possesses robust male-specific regulatory activity in the A5 and A6 segments, (B’) whereas the *D*. *ananassae* sequence has little-to-no abdominal regulatory activity. (A” and B”) EGFP-reporter transgene expression driven by sequences orthologous to the *D*. *melanogaster yellow* wing/body element in transgenic male pupae at ~85 hours after puparium formation. (A”) The *D*. *kikkawai* sequence retains the posterior stripe regulatory activities characteristic of the wing element but lacks the body element’s male-specific activity. (B”) The *D*. *ananassae* sequence possesses the regulatory activities characteristic of the wing element and body element. Red arrowheads indicate segments in which the regulatory activity is lacking.

Male-specific pigmentation has been lost several times within the *melanogaster* species group [23]. We sought to trace the paths by which this has occurred independently at the network level in two monomorphic species, *D*. *kikkawai* and *D*. *ananassae* (Fig 1). Previously, the *D*. *kikkawai* yBE was found to lack regulatory activity due to *cis*-evolution, in which a key binding site for Abd-B was lost [23]. Our *in situ* hybridization results confirm that *yellow* expression is indeed absent in the abdomen of *D*. *kikkawai* (Fig 1D”). We were curious whether the expression of *tan* had similarly been lost through *cis*-regulatory changes to its CRE. Surprisingly, *tan* expression was detected in the A6 body segment of *D*. *kikkawai* males, in a pattern reminiscent of *D*. *auraria*, a second *montium* species (Compare Fig 1C’ and 1D’), and male-limited like that seen for the expanded expression in males of species from the outgroup Oriental clade (*D*. *melanogaster*) and *ananassae* subgroup (*D*. *malerkotliana*) (Fig 1B’ and 1E’). Consistent with the endogenous expression pattern, the *D*. *kikkawai* t_MSE drove robust expression in the A5 and A6 segments of transgenic *D*. *melanogaster* pupae (Fig 3A’). These results indicate that the loss of pigmentation in *D*. *kikkawai* has proceeded without altering the ancestral expression of *tan*, suggesting that evolution of this trait occurred through other genes.

For *D*. *ananassae*, we found that the loss of male pigmentation was accompanied by the loss of *yellow* and *tan* expression in males (Fig 1F’ and 1F”). Interestingly, the CRE targets for *cis*- and *trans*-evolution were distinct from the case of *D*. *kikkawai*. Specifically, the orthologous *yellow* 5’ regulatory region retained regulatory activity in the male A5 and A6 segments in transgenic *D*. *melanogaster* (Fig 3B”), whereas the t_MSE lacked activity (Fig 3B’). As the most recent common ancestor of the *melanogaster* species group likely possessed a male pattern of abdomen pigmentation (Fig 1, node 3) [23], our results indicate that dissimilar modifications to this ancestrally dimorphic abdominal pigmentation network were responsible for these similar morphological outcomes. These divergent evolutionary paths may reflect the use of *trans*-regulatory inputs that differ between the yBE and t_MSE CREs. In order to understand how the evolution of *tan* and *yellow* expression has been individualized, we sought to characterize the regulatory linkages that control these CREs in *D*. *melanogaster*.

### Distinct combinations of Hox factors and co-factors control the coordinated activities of the t_MSE and the yBE

Previously, Abd-B was shown to be a direct activator of *yellow* expression in the A5 and A6 segments through its interaction with two binding sites in the ~1.6 kb yBE reporter (S1G Fig, vertical blue lines) [23]. With our ultimate goal being to functionally characterize the regulatory inputs responsible for *yellow* expression in the abdominal epidermis; we sought to define a more minimal CRE sequence capable of directing robust expression in the male A5 and A6 abdominal segments (S1H Fig). Thus, we created three progressively truncated forms of the yBE centered on the two Abd-B binding sites (S1G Fig). The 1.1 and 0.9 kb sequences each drove robust but ectopic EGFP reporter gene expression (S1I and S1J Fig). The third truncated version of 0.6 kb drove reporter expression in a pattern limited to the A5 and A6 segments (S1K Fig). We refer to this sequence as yBE0.6, and this was the sequence that we chose to further characterize.

**Fig 4:**
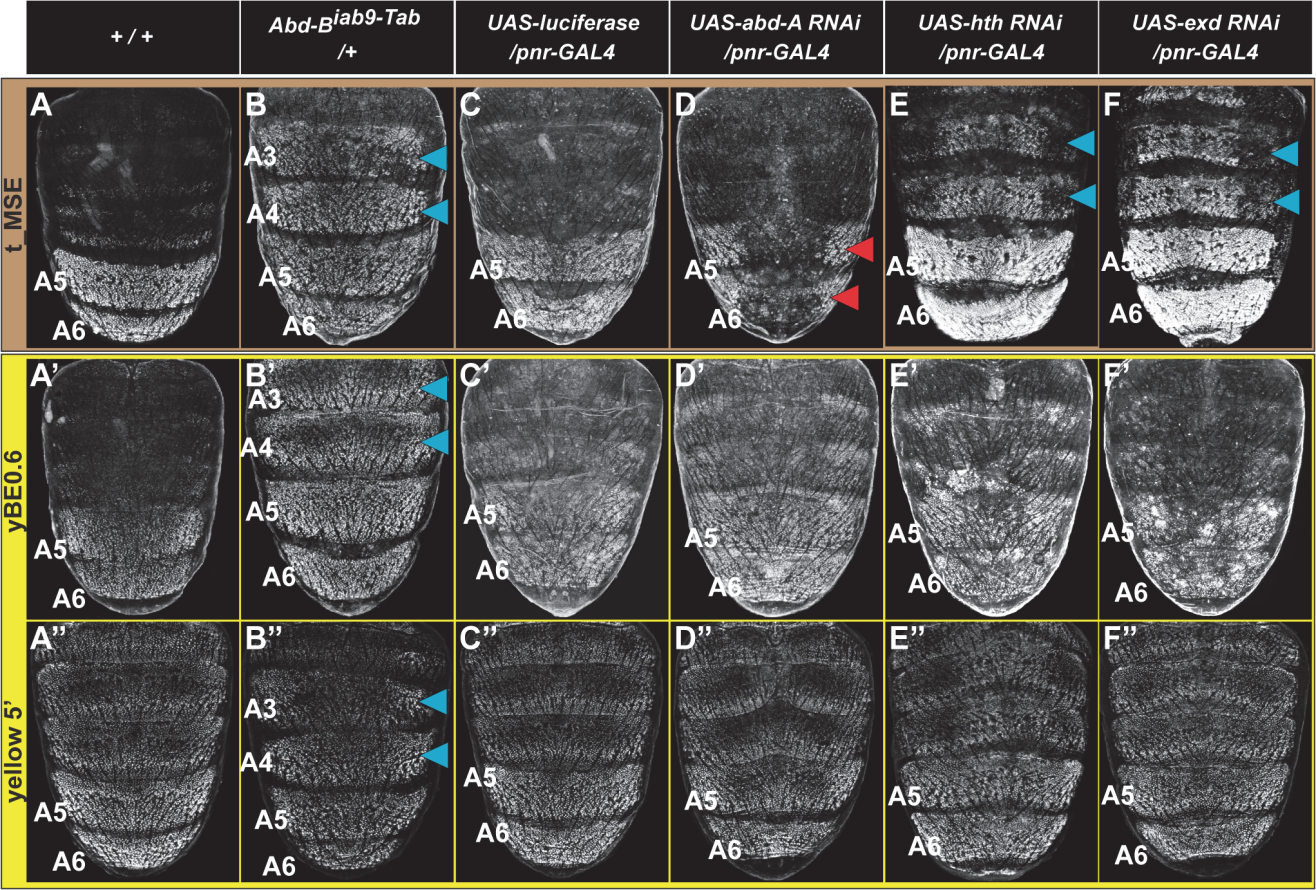
Genetic interactions between pigmentation network transcription factors and CREs regulating abdominal *tan* and *yellow* expression. (A-F) EGFP reporter expression driven by the t_MSE was imaged at ~95 hours after puparium formation in male pupae. (A’-F’) EGFP reporter expression driven by the yBE0.6 was imaged at ~85 hours after puparium formation in male pupae. (A”-F”) EGFP reporter expression driven by the *yellow* 5’ sequence was imaged at ~85 hours after puparium formation in male pupae. Genotypes altering the genetic background are listed at the top of each column. Specimens are (A, A’, and A”‘) homozygous and (B-F, B’-F’, and B”-F”) hemizygous for the EGFP reporter transgene. (B) t_MSE, (B’) yBE0.6, and (B”) *yellow* 5’ regulatory activity expands into the A3 and A4 segments where *Abd-B* is ectopically expressed. Compared to a (C) control genetic background, the (D) t_MSE regulatory activity is dramatically reduced in the midline region where *abd-A* expression is suppressed. Suppression of (E) *hth* and (F) *exd* expression results in ectopic t_MSE regulatory activity in the A4 and A3 segments. Suppression of (D’) *abd-A*, (E’) *hth*, and (F’) *exd* expression has little-to-no effect on yBE0.6 regulatory activity compared to the (C’) control genetic background. Suppression of (D”) *abd-A* and (F”) *exd* has little-to-effect on the *yellow* 5’ regulatory activity compared to the (C”) control genetic background. Suppression of (E”) *hth* results in a mild expansion of regulatory activity into the A3 and A4 segments. Blue arrowheads indicate segments where the genetic background modification resulted in ectopic reporter transgene activity. Red arrowheads indicate segments where the genetic background modification resulted in a loss of reporter transgene activity.

The coordinated patterns of *tan* and *yellow* expression in the A5 and A6 segments may be explained through the yBE0.6 and t_MSE possessing the same regulatory inputs and equivalent regulatory activities, or alternatively by these two CREs possessing unique regulatory inputs. The only known direct regulator of a pigmentation gene CRE is Abd-B’s interaction with the yBE. Genetic evidence for this regulatory input can be seen in the *Transabdominal* (*Tab*) genetic background, where ectopic *Abd-B* expression occurs in the A4 and A3 segments [35]. Consistent with *Abd-B* functioning as an upstream *trans*-activator of the yBE0.6, EGFP expression occurred ectopically in the male A4 and A3 segments of *Tab* mutants (Fig 4B’, blue arrowheads). When the t_MSE reporter gene was evaluated in the *Tab* background, a similar expansion of regulatory activity was seen (Fig 4B, blue arrowheads), suggesting that like *yellow*, *Abd-B* is an upstream activator of *tan* expression.

During development the Hox genes *abd-A* and *Abd-B* are both required to specify the identities of the A5 and A6 segments [36,37]. While Abd-B expression occurs in both the A5 and A6 segments [38], the range of Abd-A expression includes the A2-A6 segments [39]. Previously we found that Abd-A is expressed in and required for the male-specific pattern of pigmentation and t_MSE activity in the A5 and A6 segments [39]. It seemed plausible that Abd-A and Abd-B are part of a shared Hox-regulatory circuit that directs the coordinated expression of *tan* and *yellow*. To test this possibility, we used the *pnr-GAL4* chromosome to drive dorsal midline expression of an RNA interference (RNAi) transgene that specifically silences *abd-A* expression. In this genetic background, EGFP expression driven by the t_MSE was markedly reduced compared to a control genetic background in which a luciferase transgene was ectopically expressed (compare Fig 4C and 4D). To our surprise, EGFP expression driven by the yBE0.6 was not noticeably altered in the *abd-A* silenced genetic background compared to the control genetic background (compare Fig 4C’ and 4D’). These outcomes indicate that *abd-A* functions in the abdomen as an upstream regulator of *tan*, but has little-to-no effect on *yellow* expression as directed by the yBE0.6 CRE. Hence, the regulatory wiring responsible for coordinated expression of *tan* and *yellow* seem to substantially differ.

The *in vivo* selectivity of Hox proteins for their target gene CREs has been found in several cases to be enhanced through cooperative binding with Hox cofactor proteins [40]. For *Drosophila*, the best studied Hox cofactors are the transcription factors Hth and Exd. RNAi-mediated suppression of *hth* and *exd* results in ectopic pigmentation of the male A3 and A4 abdominal segments, suggesting that these Hox cofactors operate as upstream repressors of male tergite pigmentation in the A3 and A4 abdomen segments [39]. Moreover, RNAi-mediated suppression of *hth* and *exd* in the dorsal abdomen midline results in ectopic regulatory activity for the t_MSE in the male A3 and A4 segments (Fig 4E and 4F, blue arrowheads). In contrast, we found that the yBE0.6 failed to drive a comparable expanded reporter gene expression upon RNAi knockdown of *hth* or *exd* expression (Fig 4E’ and 4F’), highlighting that the coordinated activation of the yBE0.6 and t_MSE occur through distinct mechanisms that differ in Hox co-factor dependence.

We were concerned that the different utilizations of Hox and Hox cofactor inputs for the t_MSE and yBE0.6 might be an artifact of truncating down the full *yellow* regulatory sequence to a minimal element lacking key regulatory inputs. Thus, we evaluated a larger *yellow* gene 5’ sequence which contains both the wing element and body element in the same genetic backgrounds where Hox and Hox cofactor expression were modified. This larger sequence had ectopic expression in the *Abd-B* misexpression background as seen for the yBE0.6 element (compare Fig 4B” to 4B’). Like the yBE0.6 element, little-to-no change in reporter expression was observed in the *abd-A* and *exd* RNAi background (Compare Fig 4D” and 4F” to 4D’ and 4F’), results that contrast with the prominent alteration that occurs for t_MSE-directed reporter expression (Fig 4D and 4F). The only difference we observed was a modest up-regulation of *yellow* 5’-driven reporter expression in the A3 and A4 segments of the *hth* RNAi background. This suggests that *yellow* expression is responsive to *hth* through some regulatory sequence outside of the yBE0.6 element, perhaps through the wing element which drives a low level of expression in the epidermis [27].

### Spatial-mapping of yBE0.6 and t_MSE regulatory inputs

**Fig 5:**
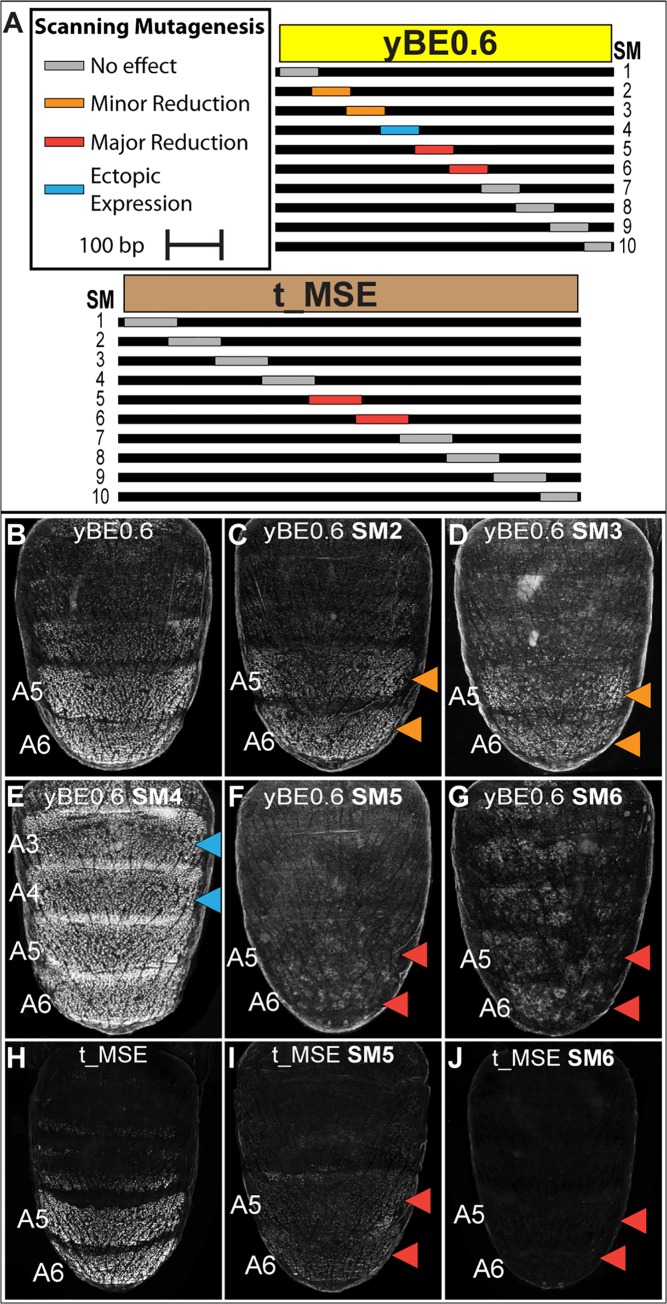
Scanning mutagenesis identifies CRE sequences required for *yellow* and *tan* expression. (A) Ten scanning mutant versions, SM1-SM10, of the *D*. *melanogaster* yBE0.6 sequence and t_MSE sequence were created. In each mutant, a block of ~70 base pairs was altered such that every other nucleotide was altered by a non-complementary nucleotide transversion. (B-G) EGFP reporter transgene expression in *D*. *melanogaster* male pupae at ~85 hours after puparium formation. (B) The yBE0.6 sequence drives reporter expression in the male A5 and A6 segments. (C and D) The SM2 and SM3 mutations resulted in a modest reduction in regulatory activity, whereas the (F and G) SM5 and SM6 mutations resulted in a near-total loss of reporter activity. (E) The SM4 mutation led to a pan-abdomen increase in regulatory activity. (H-J) EGFP reporter transgene expression in *D*. *melanogaster* male pupae at ~95 hours after puparium formation. (H) The t_MSE sequence drives expression in the male A5 and A6 segments. (I and J) The SM5 and SM6 mutations resulted in a loss of reporter expression in the male abdomen. Red arrowheads indicate regions where the regulatory activity was greatly reduced due to a scanning mutation and the Orange arrowheads indicate regions where regulatory activity was modestly reduced. Blue arrowheads indicate regions where regulatory activity was gained due to a scanning mutation.

In order to more comprehensively characterize the regulatory linkages within the yBE0.6, we created 10 scanning mutant (SM1—SM10) versions of the yBE0.6 (S5 and S6 Fig). For each scanning mutant, a single contiguous block of ~70 bp was altered at every other base pair to its non-complementary transversion (S5 Fig). The regulatory activity characteristic of the yBE0.6 was not notably altered by the SM8 and SM9 mutations. For several scan mutants, activity was either reduced (Fig 5C and 5D; SM2 and SM3) or lost (Fig 5F and 5G; SM5 and SM6), indicating that the mutated blocks likely encode binding sites for activating transcription factor inputs. The SM5 and SM6 mutations spanned CRE sequences that include the bona fide binding sites for Abd-B [23], though we left these binding sites unaltered in these two scanning mutants (S5 Fig). The diminished regulatory activity caused by both SM5 and SM6 indicates that Abd-B collaborates with other adjacent binding transcription factors to activate gene expression in the pupal abdomen. The reduced regulatory activity of the SM2 and SM3 mutants demonstrates that additional activating inputs reside outside of the known Abd-B sites.

In addition to scanning mutations resulting in reductions in yBE0.6 regulatory activity, three resulted in gains in regulatory activity suggesting that the mutated sequences disrupted binding sites for repressive transcription factor inputs. yBE0.6 regulatory activity in the male A2-A6 abdomen segments was notably increased by the SM4 alteration (Fig 5E). The yBE0.6 SM8 and SM10 CREs each exhibited a modest ectopic regulatory activity in the male A4 and A3 segments (S6I and S6K Fig). Collectively, these results suggest that repressing and activating inputs are distributed throughout the 660 base pairs of yBE0.6.

A similar scanning mutagenesis strategy was carried out for the 860 bp t_MSE (Fig 5A, and S7 and S8 Fig), in which each scanning mutation spanned ~80 bp. While the t_MSE regulatory activity was not noticeably altered in 8 of 10 scanning mutants (S8 Fig), the SM5 and SM6 alterations each resulted in a dramatic reduction of EGFP reporter expression in the male A5 and A6 segments (Fig 5I and 5J). In order to more precisely localize activation inputs, we generated a series of fine-scale scanning mutations (S9 and S10 Fig) within a minimal 351 bp subfragment of the t_MSE that reproduces its activity (“t_MSE2”, S1 Fig). For the 351 bp t_MSE, we generated 10 scanning mutants in which each mutation spanned ~20 bp and collectively covered the entire SM5 and SM6 region (S9 Fig). While the 5i1, 5i3, 5i4, and 6i1–6i3 scanning mutants had no noticeable effect on t_MSE2 regulatory activity, scanning mutants 5i2, 6i4, 6i5, and 6i6 consistently drove reduced reporter expression in the male A5 and A6 segments (S10 Fig). Thus, this CRE has activating inputs located within the SM5 and SM6 regions. We sought to determine if these activating inputs include binding sites for Abd-A and Abd-B.

### The t_MSE is an indirect and direct target for posterior Hox proteins

Abd-B is a key direct regulatory input for the yBE that is necessary to drive *yellow* expression throughout the male A5 and A6 segments [23], and yet, the yBE has little-to-no response to alterations of Abd-A. To disentangle how *tan* generates a correlated pattern with *yellow*, but is genetically downstream of both Abd-B and Abd-A, we sought to determine whether these Hox factors directly bind the t_MSE. An *in vitro* study has demonstrated a preference of TTAT sites for Abd-B binding and TAAT sites for Abd-A [41]. Within the SM5i2 region required for A5 and A6 regulatory activity, resides only a single TTAT site (S9 Fig). However, this site also occurs in the overlapping region for the SM5i1 region which is dispensable for abdominal activity as this mutant CRE has 114±3% of the wild type CRE’s activity in the male A5 segment (S10D Fig). Thus, we did not further consider the SM5i2 region as a location for direct Hox-regulation.

**Fig 6:**
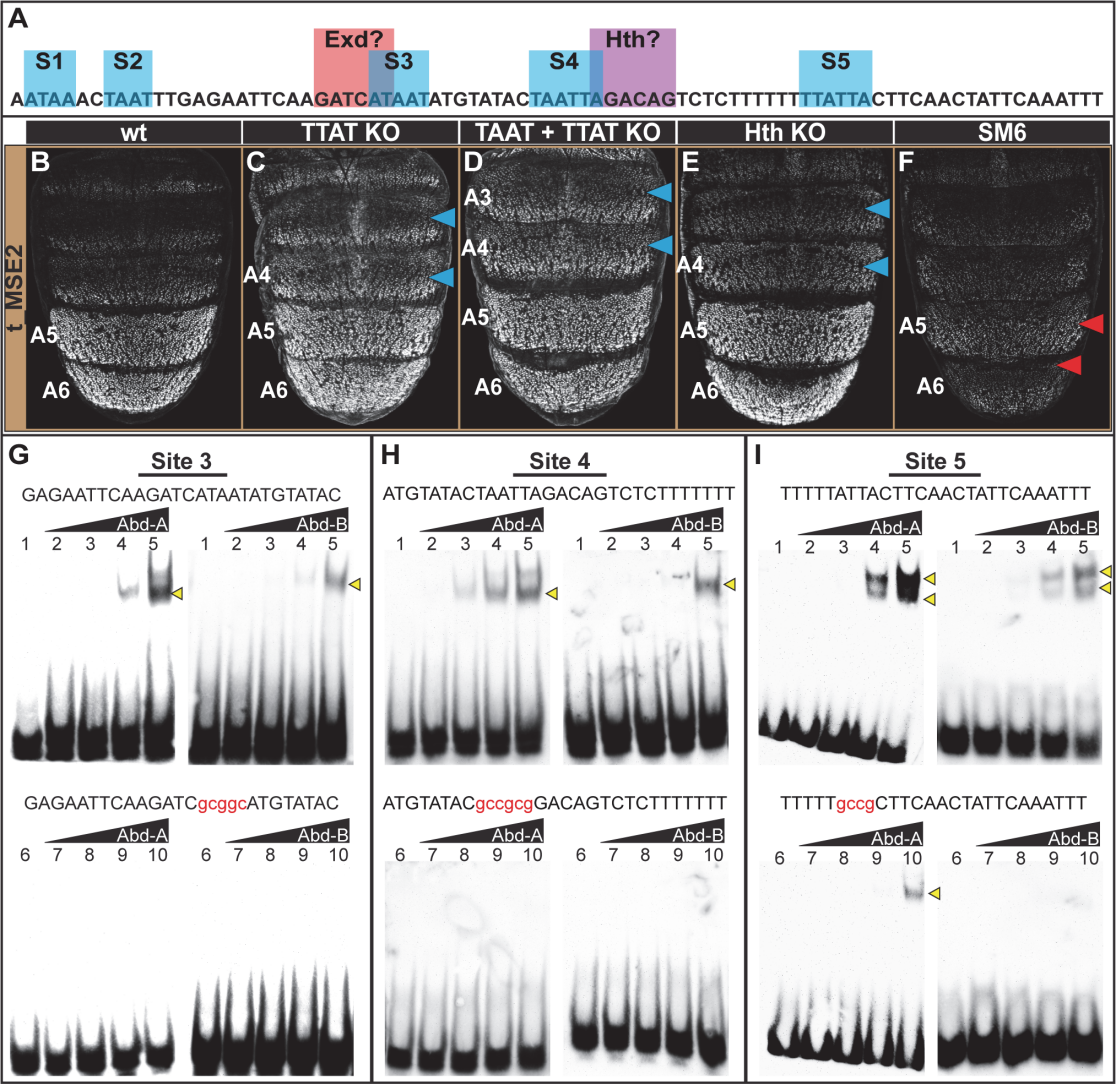
Characterization of the direct Hox inputs shaping *tan* expression. (A) The SM6 region of the t_MSE possesses five sites, S1-S5, with sequences characteristic of Abd-B (TTAT) and Abd-A (TAAT) binding sites. This CRE region also possesses sites resembling sequences bound by Exd and Hth, though the functionality of the Exd site was not studied here. (B-F) EGFP reporter transgene expression in male pupae at ~95 hours after puparium formation. (B) The t_MSE2 sequence drives robust expression in the male A5 and A6 segments. When all of the (C) TTAT sites and (D) TTAT and TAAT sites depicted in (A) were mutated, regulatory activity in the male A5 segment was reduced to 89±6% and 88±3% respectively. (F) When the entire SM6 region was mutated, regulatory activity decreased to 31±1%. (C) The TTAT site mutations resulted in activity increasing in the A4 and A3 segments respectively to 169±4% and 261±6%. (D) The TTAT and TAAT site mutations resulted in activity increasing in the A4 and A3 segments respectively to 207±2% and 281±2%. (E) When the Hth site was mutated, regulatory activity in the male A5, A4, and A3 segments respectively increased to 122±7%, 236±6%, and 276±4%.(F) The entire SM6 region mutation resulted in activity decreasing in the A4 and A3 segments respectively to 64±1% and 73±1%. Blue arrowheads indicate segments where activity was notably increased and Red arrowheads indicate segments with notably decreased activity compared to the wild type sequence. Gel shift assays for sequences possessing wild type and mutant site (G) 3, (H) 4, and (I) 5 and the DNA-binding domains for Abd-A and Abd-B. Binding reactions used increasing amounts of the GST-DNA binding domain fusion protein (from left to right: 0 ng, 111 ng, 333 ng, 1000 ng, and 3000 ng). Binding correlated with the amount of input protein for the probes with the non-mutant sequence, whereas binding was dramatically reduced for the probes with a mutant Hox site.

We next considered the SM6 region, for which the scanning mutation (SM6) resulted in a drastic reduction of male activity to 31±1% of the wild type CRE (compare Fig 6B and 6F). Within this 85 bp SM6 region resides 5 sequences matching either TTAT or TAAT sites, or both (Fig 6A, sites 1–5 or S1-S5). We generated and tested two mutant t_MSE2 reporter constructs, one in which the three TTAT sequences were mutated and the other for which the TTAT and TAAT sites were mutated (S11 Fig). To our surprise, the activity of the TTAT site mutant was only modestly reduced to 89±6% of the wild type CRE in the A5 segment (compare Fig 6B and 6C). Moreover, when the two TAAT sites were additionally mutated, regulatory activity was measured at 88±3% of wild type (Fig 6D). This lack of a pronounced regulatory effect contrasts with that caused by the 85 bp scanning mutation 6 (Fig 6F). Thus, with respect to the posterior male segment activity of the t_MSE2, it appears that Abd-B and Abd-A play little-to-no role as direct activators.

While the TTAT and TAAT sites had little-to-no importance regarding the activity of the t_MSE2 in the A5 and A6 segments, these sites were important for limiting reporter expression to these more posterior abdomen segments. When the TTAT sites were mutated, the regulatory activity in the A4 and A3 segments respectively increased to 169±4% and 261±6% of the wild type sequence (Fig 6C, blue arrowheads). When all TTAT and TAAT sites were mutated, more pronounced increases in regulatory activity were observed in the A4 (207±2%) and A3 (281±2%) segments (Fig 6D, blue arrowheads). These effects contrast with the modest reductions in activity that occurred for the scanning mutation 6 (Fig 6F; 64±1% for A4 and 73±1% for A3). Our results suggest that these sequences function as Hox binding sites to repress *tan* expression in abdominal segments anterior to that of A5. Of note, these anterior segments express Abd-A, but not Abd-B. Hence, unlike the yBE, the t_MSE appears to have co-opted direct Abd-A regulation in a unique way.

To further address whether Abd-A directly binds to and regulates the t_MSE, we focused our attention on sites S3, S4, and S5, as these sites were closely associated with a sequence resembling a site for Exd and one resembling a site for Hth (Fig 6A). We found that Abd-A bound to probes with the wild type sites but little-to-no binding occurred with the mutant versions (Fig 6G–6I), indicating that Abd-A can specifically bind to these sequences. A similar specific binding was shown for Abd-B, suggesting that these sequences can also be bound by this Hox protein.

It seemed plausible that the adjacent cofactor sites might be necessary to repress t_MSE activity in the anterior abdominal segments in conjunction with Abd-A. As a cursory test of this possibility, we mutated the putative Hth-site within the context of the t_MSE2 sequence (S11 Fig) and tested this sequence’s capability to regulate EGFP expression in transgenic pupa (Fig 6E). Consistent with this site being necessary for repression, the regulatory activity in the A3 and A4 segments respectively increased to 236±6% and 276±4% of the wild type sequence (Compare Fig 6B to 6E). Collectively, the t_MSE’s abdominal regulatory activity occurs through an encoding distinct from that responsible for the similar pattern of *yellow* expression directed by the yBE0.6 CRE.

## Discussion

**Fig 7:**
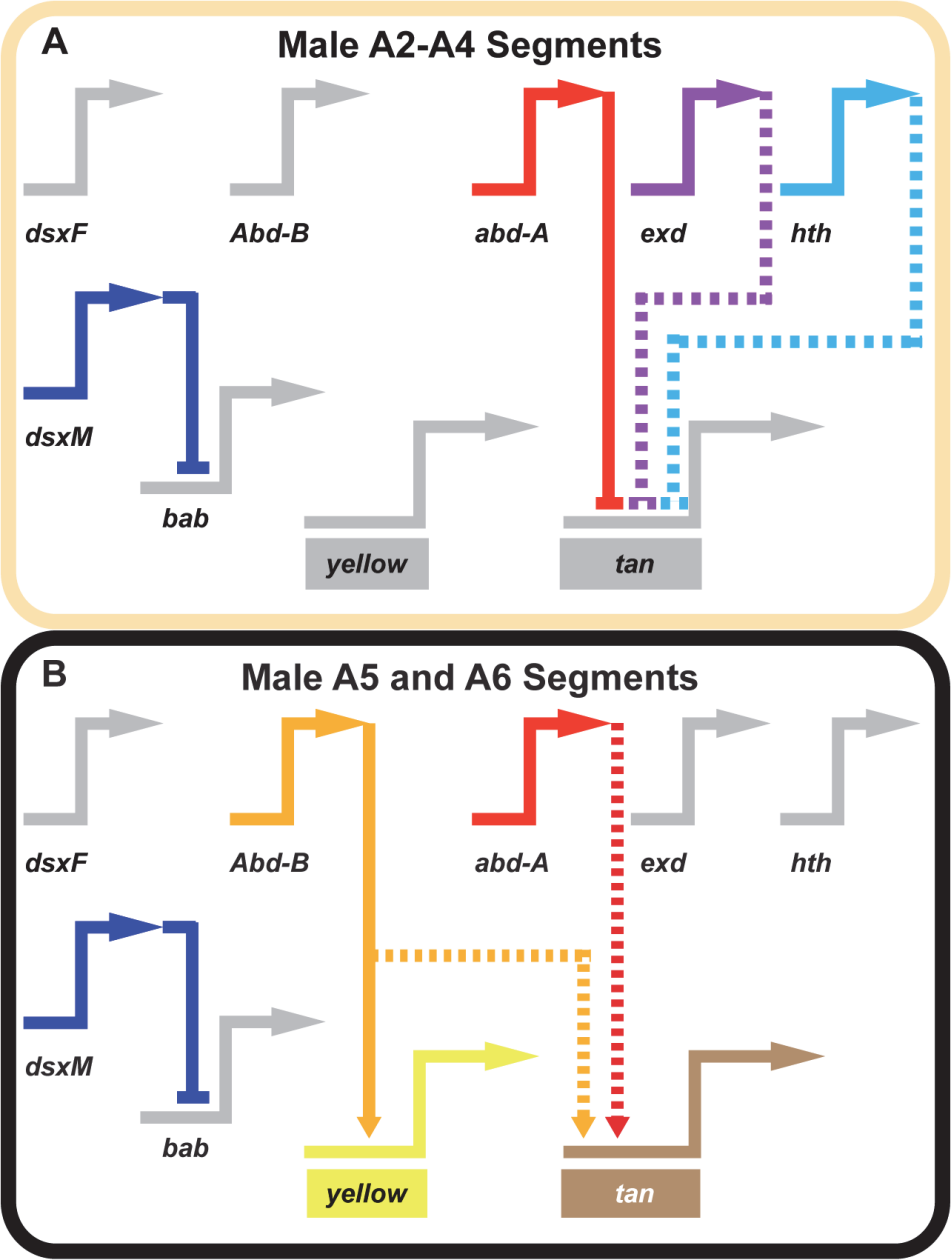
Gene network models for unpigmented and pigment abdominal segments. Wiring diagram of pigmentation gene networks experienced by the (A) non-melanic male A2-A4 segments and (B) the melanic A5 and A6 segments. (A) Abd-B expression is lacking in the anterior A2-A4 segments and as a result *yellow* and *tan* lack the direct and indirect activating input from this transcription factor. In these segments, Abd-A forms direct repressive inputs with *tan* which are supported (directly or indirectly) by the repressive effects of *exd* and *hth*. (B) Abd-B is expressed in the posterior A5 and A6 segments, where it acts as a direct activator of *yellow* and an indirect activator of *tan*. In these segments, Abd-A acts as an indirect activator of *tan* expression as well. In these schematics, inactive genes are indicated in gray coloring, solid connections between genes indicate validated direct interactions between a transcription factor and a pigmentation gene CRE, and dashed connections indicate indirect interactions or those not yet shown to be direct. Connections terminating with an arrowhead indicate connections in which the transcription factor functions as an activator, and connections terminating in a nail head shape indicate a repressive relationship.

Here, we have traced the evolutionary history of two CREs required for a novel trait, and show that they have recently evolved similar expression patterns through remarkably different architectures in a common *trans-*regulatory landscape. Our data indicates that the tergite-wide activities of the yBE and t_MSE did not exist in the monomorphic ancestor for *Sophophora*, but evolved in the lineage leading to the common ancestor of the *melanogaster* species group. Our results support a scenario where the subsequent expansion and contraction of male pigmentation pattern was driven primarily by alteration of the *trans*-regulators, whereas repeated losses involved both *cis*- and *trans*-evolution with respect to these CREs. Though the t_MSE and yBE drive coordinated patterns of gene expression, we found striking differences in their upstream regulators and direct regulatory linkages (Fig 7). These results bear on our understanding of how new gene regulatory networks form, diversify, and how coordinated regulatory activities can arise through the independent evolution of unique regulatory codes.

### Inferring a mechanism for a nascent Hox-regulated genetic switch

Hox transcription factors play a prominent role in generating the differences in serially homologous animal body parts, and the origin of novelties [42]. The diversification of homologous parts can be driven by changes in the spatial domains of Hox protein expression, as has been shown for crustacean appendage morphology [43], snake limblessness [44], and for the water strider appendage ground plan [45]. Changes in the downstream Hox targets are evident in cases such as the hindwings of insects [46], and for fruit fly tergite pigmentation [23]. The origin of novel structures can also be traced to the co-option of Hox proteins, as exemplified by cases such as the *Photuris* firefly lantern [47] and the sex combs residing on the forelegs of certain *Drosophila* species [48,49]. For many of these evolved traits, the molecular mechanisms by which Hox expression patterns and target genes evolve remain unknown.

While mechanistic studies on the evolution of Hox-regulated CREs remain limited, several target gene CREs have been thoroughly characterized and serve as exemplars of Hox-regulation during development [40]. Hox proteins can interact with CRE binding sites as monomers [50] or through cooperative interactions with Hox-cofactors [51–53]. The activity of these bound complexes can be further modulated through interactions with collaborating transcription factors. However, to date, few direct Hox target linkages have been traced to their evolutionary beginnings. Expression of *yellow* in the male A5 and A6 segments required the gain of two binding sites for Abd-B [23], but it remains uncertain whether these binding events require cooperative interactions with Hox cofactors and which transcription factors are acting as collaborators.

The t_MSE presented an opportunity to study how a second Hox-responsive CRE evolved in parallel to the activity at *yellow*. In this study, we show that Abd-A and Abd-B respectively are necessary and sufficient for t_MSE regulatory activity. However, we show that the ablation of the resident Hox sites had little effect on this CRE’s activity in the A5 and A6 segments, though mutations to nearby CRE sequences resulted in dramatically reduced activity. This result strongly implies that both Abd-A and Abd-B indirectly activate the t_MSE through a downstream factor or factors. While it can’t be entirely ruled out that these factors are operating directly through other non-canonical Hox sites, our gel shift assays did not provide convincing evidence that such sites exist. While the Hox sites were not necessary for activation in the A5 and A6 segments, their ablation resulted in a drastic gain of regulatory activity in the A4 and A3 segments, a setting in which Abd-A is the only Hox protein present. This indicates that Abd-A is a direct repressor of t_MSE function in these anterior abdomen segments. The observed dichotomy in Abd-A function can be explained by at least two—not necessarily mutually exclusive—scenarios. First, in the A5 and A6 segments Abd-B may not act as a direct activator of the t_MSE but its occupancy of Hox sites might preclude the direct repressive effects of Abd-A. Secondly, Abd-A may interact cooperatively or collaboratively with other transcription factors in the more anterior segments to impart repression. Our results with Hth support this second scenario.

The Hox co-factors Hth and Exd were prime candidates to mediate the context-dependent modulation of Abd-A activity. First, RNAi suppression of *hth* and *exd* expression each resulted in ectopic pigmentation [39] and t_MSE activity in the male A4 and A3 segments (Fig 4). Furthermore, inspection of the t_MSE sequence revealed sites characteristic of Hth (AGACAG) and Exd (GATCAT) binding that reside in close proximity to Hox sites (Fig 6A). This site content and arrangement is strikingly similar to that found in an abdominal-repressive module for the CRE controlling thoracic *Distalless* expression [51,54]. Along a similar vein, we show that the ablation of the Hth-like site led to an anterior expansion in t_MSE activity similar to that induced by the Hox site mutations (Fig 6). This outcome supports the interpretation that the more recent origin of the t_MSE involved the formation of novel regulatory linkages with Hox proteins and Hox cofactors.

### The origins of a network controlling a sexually dimorphic trait

Morphological traits result from the activities of gene regulatory networks, in which each network is governed by a *trans*-regulatory tier of transcription factors and cell signaling components that ultimately regulate the expression of a set of differentiation genes [1,55,56]. For animals, the *trans*-regulatory genes are remarkably conserved [2,57]. It is plausible that the origin of new morphologies occurs through the formulation of new gene regulatory networks, while diversification and losses in traits would likely occur through the modification and dismantling of extant networks. The empirical evaluation of such trends of network evolution necessitates the study of trait evolution at the level of networks, CREs, and their encoded binding sites for multiple animal lineages, traits, and evolutionary time frames. The *Drosophila* pigmentation system is particularly well poised to make pioneering contributions to this growing body of knowledge.

The most recent common ancestor of monomorphic and dimorphic *Sophophora* lineages was inferred to have possessed monomorphic tergite pigmentation (Fig 1, node 1) [23], in the context of an otherwise invariant morphological landscape, in which segment number and form has remained conserved at the genus level. Hence, the origin of this novel pigmentation trait may be expected to have co-opted spatial and sex-specific patterning mechanisms that shape the conserved abdomen features. Our comparative analysis of orthologous *yellow* and *tan* non-coding sequences indicate that these co-option events involved the origination of novel CRE activities that connected a *trans*-regulatory tier of Hox, Hox-cofactors, and the Bab proteins to these key differentiation genes that encoded pigmentation enzymes (Fig 7).

The patterns of regulatory activity for the orthologous *tan* and *yellow* sequences support some additional inferences about the early events in this dimorphic trait’s origin. While the t_MSE abdominal activity was strikingly lower in *D*. *pseudoobscura* and *D*. *willistoni*, the *D*. *pseudoobscura yellow* body element was active (albeit with expanded activity). These outcomes support at least two evolutionary scenarios. One scenario is a sequence of events where the origination of the t_MSE and y_BE in the lineage of *D*. *pseudoobscura* (Fig 1, node 2) was followed by a secondary loss of the t_MSE. This scenario is supported by our previous observation of dimorphic Bab expression in the *D*. *pseudoobscura* abdomen [33], backing the notion that this species’ broad pattern of monomorphic abdominal pigmentation evolved from a dimorphic ancestral state. For the other scenario, the body element-like regulatory activity of *D*. *pseudoobscura* could be due to this CRE’s origin preceding (Fig 1, node 2) that of the t_MSE (Fig 1, node 3). Distinguishing between these two scenarios will require a more rigorous comparison of the pigmentation phenotypes and networks within the *melanogaster* and *obscura* species groups. The outcomes would provide a more nuanced understanding of the early evolutionary history for the derived sexually dimorphic pigmentation network.

### Diversification and deconstruction of sexually dimorphic pigmentation

Tergite pigmentation evolution in the *Sophophora* subgenus has been relatively well-studied, and the accumulated results frame an extended perspective of trait evolution within a common network (Table 1). *Trans*-evolution at the *bric-à-brac* (*bab*) locus has been found to be a major driver for the diversification of female tergite pigmentation [16,58,59]. This study, in addition to previous studies, indicates that *trans*-evolution at as of yet unidentified loci may have played prominent roles in the diversification of male-limited tergite pigmentation [26,30]. Regarding the repeated losses in male pigmentation, our results are consistent with a scenario where both *trans*- and *cis*-evolution occurred, though the targets of *cis*-evolution have alternated between *tan* and *yellow* [23,26]. While *cis*-evolution has been identified for a case of monomorphic gain (*ebony*) in tergite pigmentation [8], and for a case of monomorphic loss (*ebony* and *tan*) [60], the full wealth of case studies portend to a more prominent role for evolutionary changes in the *trans*-regulatory tier of the pigmentation gene network. However, it is important to note that many of these case studies only assessed the activities of transgenes in *D*. *melanogaster*. While similarities in CRE activity might be indicative that expression divergence occurred through *trans*-evolution, it does not rule out the possibility that *cis*-changes occurred at other regions in the pigmentation enzyme gene loci, or that expression divergence results from combined *cis*- and *trans*-changes. In the future, it will be important to validate or reject the prominent role for *trans*-regulatory evolution by the reciprocal tests of CREs in species with the contrasting patterns of pigmentation. Two studies where CREs were tested in species with contrasting pigmentation phenotypes, showed that *trans*-regulatory evolution was a major driver for diversification of fruit fly wing spot patterns by modifying Distalless and wingless expression [10,61]. Thus it appears the notion of a “conserved *trans*-landscape” requires more scrutiny.

**Table 1:** A pigmentation enzyme gene perspective of network evolution.

**Trait (sex affected)**	**Species**	**Divergence**	**Change(s)**	**Reference**
Increased Tergite Pigmentation	D.melanogaster	Intraspecific	cis (ebony)	Rebeiz et al. 2009
Increased Tergite Pigmentation (F)	D.melanogaster	Intraspecific	trans (bab) trans (bab) trans(bab) and cis (tan)	Kopp et al. 2003 Rogers et al., 2013Bastide et al., 2013
Dark/Light Tergite Pigmentation (F)	D. yakuba D. fuyamai	Interspecific	trans (bab)	Rogers et al., 2013
Loss of Tergite Pigmentation (M)	D. santomea	Interspecific	trans (?) cis (tan)	Jeong et al., 2006 Jeong et al., 2008
Loss of Tergite Pigmentation (M)	D. kikkawai	Interspecific	cis (yellow)	Jeong et al., 2006
Loss of Tergite Pigmentation (M)	D. ananassae	Interspecific	trans (?) cis (tan)	This study
Expansion of Tergite Pigmentation (M)	D. prostipennis	Interspecific	trans (?) cis (yellow)	Ordway et al., 2014
Expansion of Tergite Pigmentation (M)	D. malerkotliana	Interspecific	trans (?)	This study
Retraction of Tergite Pigmentation (M)	D. auraria	Interspecific	trans (?)	This study
Gain of Sexual Dimorphism	melanogaster group	Interspecific	trans (bab) cis (yellow) and (tan)	Williams et al., 2008 This study
Light Body Coloration	D. novamexicana	Interspecific	cis (tan and ebony)	Wittkopp et al., 2009
Gain of wing spot	oriental lineage	Interspecific	cis (yellow)	Gompel et al., 2005 Arnoult et al., 2013
Loss of wing spot	oriental lineage	Interspecific	cis (yellow)	Prud’homme et al., 2006
Diversification of wing spot	oriental lineage	Interspecific	trans (Dll)	Arnoult et al., 2013
Novel wing spots	D. guttifera	Interspecific	cis (yellow) trans (Wg)	Werner et al., 2010

In this study, and elsewhere, experiments indicate that pigmentation losses are associated with and perhaps result from both changes in the *trans*-regulatory tier and in the *cis*-regulatory regions of the *yellow* and *tan* genes (Table 1). Interestingly, some instances of *trans*-regulatory modifications that cause loss of gene expression appear to leave perfectly good CREs intact. Our data provides a second instance in which loss of expression occurred without the loss of the encoded CRE. The yBE was found to be conserved in *D*. *santomea*, which diverged from *D*. *yakuba* ~400,000 years ago [23]. The activity for this CRE has also remained for *D*. *ananassae* since its divergence from a pigmented ancestor. In contrast, *D*. *kikkawai* has lost pigmentation while still expressing *tan* in the abdomen through a perfectly active t_MSE. These results suggest that these CREs were maintained within the population for long periods of time, perhaps indicating additional functions that promote the preservation of these CREs’ ancestral potential [62–64]. Furthermore, the observed heterogeneity of changes in *cis* and *trans* to *yellow* and *tan* were at first surprising. However, our study of the binding site architecture at the yBE and t_MSE provided key clues as to why their evolution may often be uncoupled.

### Coordinate expression patterns through discordant CREs

The coordinated expression of genes is a ubiquitous theme in developmental biology. Gene expression is finely regulated during development through the activities of CREs that are individually encoded as evolved combinations of transcription factor binding sites (regulatory logic). A compelling question is whether such synchronized expression results from the independent evolution of CREs with similar logics. This question was previously pursued for CREs of regulatory genes coordinately expressed in the developing fruit fly neurogenic ectoderm [65]. In this case, the coordinately activated CREs are encoded by a common regulatory logic, or a so called “*cis*-regulatory module equivalence class” [66]. However, the neurogenic ectoderm CREs are deeply conserved, and arose in the distant past (over 230 million years ago).

The recently evolved male-specific expression patterns for *tan* and *yellow* present a case in which the evolutionary formation of coordinated regulation can be observed over shorter time-scales. Though both the t_MSE and yBE0.6 drive reporter expression in the dorsal A5 and A6 segment epidermis of males during late pupal development, we found their regulatory logic to be surprisingly dissimilar. Whereas the yBE0.6 is directly activated by Abd-B, our results indicate that the t_MSE is indirectly activated by Abd-B and Abd-A, and is directly repressed in more anterior body segments by Abd-A and seemingly Hth. Thus, this study provides an example that illustrates how coordinated expression evolved through the evolution of very different binding site architectures and logic.

The disparity of regulatory logic governing the yBE0.6 and t_MSE sheds light on the evolutionary tendencies of gene regulatory networks. The incipient stages of the dimorphic pigmentation network’s origin involved the derivation of CREs that generate similar patterns through distinct combinations of binding sites. This evolutionary history establishes a “branched” network in which several of the possible *trans-*regulatory alterations are incapable of generating coordinated shifts in the expression patterns for co-expressed genes. Hence, an emerging theme from the work in this system is that the differences in regulatory logic of yBE and t_MSE may necessitate changes in one CRE or the other, but is unable to be altered through a common *trans* regulator that influences both CRE’s patterning. Future studies are needed to substantiate the occurrence and identity of the *trans* changes altering this network’s structure. As other recently derived morphological traits are resolved to the level of binding sites within their networks, it will be instructive to see whether similar branched networks and paths of *cis* and *trans* evolution permeate their origin and diversification. The net results may reveal general principles of gene regulatory network evolution.

## Materials and Methods

### Fly stocks and genetic crosses

Fly stocks were maintained at 25°C on a sugar food medium that was previously described [33]. CRE sequences were obtained from the *D*. *melanogaster* (14021–0231.04), *D*. *kikkawai* (14028–0561.14), *D*. *malerkotliana* (14024–0391.00), *D*. *ananassae* (14024–0371.33), and *D*. *willistoni* (14030–0811.24) species stocks from the San Diego Drosophila Stock Center, and *D*. *biarmipes*, *D*. *auraria*, and *D*. *pseudoobscura* species stocks that were obtained from Dr. Sean B. Carroll. Tests for genetic interactions with *Abd-B*, *abd-A*, *exd*, and *hth* were done using the yBE0.6-EGFP transgene that was inserted into the attP40 site on chromosome 2 [67] and the *yellow* 5’-EGFP and t_MSE-EGFP transgenes were inserted into the 51D attP site on chromosome 2 [68]. All other reporter transgenes used in this study were inserted into the attP2 site on chromosome 3 [69].

*D*. *melanogaster* stocks possessing the *Abd-B* ^*iab9Tab*^ (BDSC ID#8620) allele, and *UAS-abd-A* RNAi (BDSC ID#28739), *UAS-exd* RNAi (BDSC ID#34897), *UAS-hth* RNAi (BDSC ID#27655), UAS-luciferase control (BDSC#35788), and *pnr-GAL4* (BDSC ID#3039) transgenes were obtained from the Bloomington Drosophila Stock Center. The effects of ectopic *Abd-B* on *tan* and *yellow* loci CRE activities was observed for flies of genotype *CRE-EGFP*/*+*;*Abd-B* ^*iab9Tab*^/*+*. The effects of reduced *abd-A/exd/hth* expression on CRE activity were observed for flies of genotype *CRE-EGFP*/*+*;*UAS-*gene specific RNAi/*pnr-GAL4*. The *pnr-GAL4* stock has a chromosome where the *GAL4* gene is inserted in the *pannier* (*pnr*) locus resulting in GAL4 expression in the dorsal-medial abdomen [70].

### *in situ* hybridizations

*in situ* hybridizations were carried out as previously described [26]. Briefly, digoxigenin labeled riboprobes for *yellow* and *tan* were prepared through *in vitro* transcription of PCR templates amplified from each species (S1 Table for primers). Pupal abdomens were dissected at optimal time points for the visualization of *yellow* (70–80 h APF) and *tan* (85–95 h APF). Probe hybridization was visualized through an anti-digoxigenin antibody (Roche Diagnostics), detected by an alkaline phosphatase reaction using BCIP/NBT (Promega).

### DNA sequence alignments

The contiguous genomic DNA sequence spanning from the first exon of *Gr8a* through the last exon (exon 8) of *tan* was obtained from the *D*. *melanogaster* genome version FB2013_05. Orthologous contigs were retrieved by BLAST searches of fruit fly genomes using the *tan* exon 8 as the query sequence [71,72]. The following GenBank accessions were identified as possessing the genome sequences orthologous to *tan*: *D*. *biarmipes* (AFPP01032826), *D*. *yakuba* (CM000162), *D*. *kikkawai* (AFFH02000000), *D*. *ananassae* (AAPP01016557), *D*. *pseudoobscura* (AADE01002480), and *D*. *willistoni* (AAQB01008786). Sequences were aligned to the annotated *tan* locus of *D*. *melanogaster* using mVISTA comparative genomics tool (S2 Fig) [73]. Sequence visualizations for the *tan* and *yellow* loci of *D*. *melanogaster* (S1 Fig) were made using the GenePalette tool [74].

### EGFP reporter transgenes

EGFP reporter transgenes were used as a surrogate for the endogenous expression of the *yellow* and *tan* genes. Reporter transgene were assembled by cloning CREs into the *AscI* and *SbfI* restriction enzyme sites of the S3aG vector [75]. Each reporter transgene includes a CRE sequence cloned 5’ of a minimal *hsp70* promoter and the coding sequence of the EGFP-NLS reporter protein [76]. All reporter transgenes analyzed were integrated into an attP landing site using ϕC integrase methods (Best Gene Inc.) [69]. The primer pairs used to clone *D*. *melanogaster yellow* and *tan* CREs are presented in the S2 Table. The primer pairs used to clone orthologous CREs are presented in the S3 Table. Scanning mutant sequences for the yBE0.6 (S5..), t_MSE (S7 Fig), and t_MSE2 (S9 Fig), the t_MSE2 with putative Hox sites mutated (S11 Fig), and the *D*. *ananassae* t_MSE were synthesized by GenScript USA Inc. These synthesized sequences were flanked by an *AscI* and *SbfI* restriction enzyme sites for cloning CREs into the S3aG vector.

Quantitative comparisons of the levels of EGFP reporter gene expression driven by the t_MSE2, t_MSE2 5i1, t_MSE2 SM6, t_MSE2 TTAT sites knockouts (KO), t_MSE2 TTAT +TAAT sites KO, and t_MSE2 Hth site KO CRE sequences were performed similar to that previously described for another CRE [16,75]. For each transgene, EGFP expression was imaged from five independent replicate specimens using a confocal microscope with settings for which few pixels were saturated when EGFP expression was driven by the wild type t_MSE2. For each confocal image, a separate pixel value statistic was determined for the dorsal epidermis of the A3, A4, and A5 segments using the Image J program [77]. For each reporter transgene, the regulatory activity was calculated as the mean pixel value and standard error of the mean (SEM). Activities reported in Fig 6 were normalized to the activity for the wild type t_MSE2.

### Imaging of fly abdomens

Images of fruit fly abdomen pigmentation patterns were taken using an Olympus SZX16 Zoom Stereoscope and Olympus DP72 digital camera. Specimens were prepared for 5–10 day old adults. Projection images for EGFP-NLS reporter transgene expression were generated with an Olympus Fluoview FV 1000 confocal microscope and software. The regulatory activities of the t_MSE and t_MSE2 sequences were evaluated at ~90 hours after puparium formation (hAPF), a time point during pupal development when dimorphic expression of *tan* is first observed in the abdomen [26]. The regulatory activities for the *yellow* gene CREs were evaluated at ~85 hAPF, a time point when endogenous sexually dimorphic *yellow* expression is observed [23]. In each figure comparing CRE activities, a representative image was selected from replicate specimens (n≥6) and that were processed through the same modifications using Photoshop CS3 (Adobe). *In situ* hybridization images were taken on the same day using the same microscope and camera, and representative images were selected for Fig 1 and processed through the same modifications.

### Gel shift assays

Reverse complementary oligonucleotides were synthesized (Integrated DNA Technologies) that contain t_MSE2 sequence with wild type or mutant Hox sites (S4 Table). Each oligonucleotide was biotin-labeled on their 3’ end using the DNA 3’ End Biotinylation Kit (Thermo Scientific) and complementary oligonucleotides were annealed by standard protocol. Labeling efficiency for each binding site was determined using a quantitative Dot Blot assay (DNA 3’ End Biotinylation Kit, Thermo Scientific). Each probe was separately tested for binding with a GST-Abd-B DNA Binding Domain (DBD) [6,23] and GST-Abd-A DBD fusion proteins. The coding sequence for amino acids 136–209 of *D*. *melanogaster abd-A* was cloned 3’ to that for GST in the EcoRI and NotI sites of the pGEX4T1 vector (Amersham). This *abd-*A sequence was amplified using the primers: ACCGgaattcTGTCCACGAAGGCGCGGTCGC and AGCCgcggccgcTCATTAGCGTCGCGCCTGTTCATTTATTTCC. All gel shift reactions included 20 fmol of one labeled binding site and GST-fusion protein in General Footprint Buffer (50 mM HEPES pH 7.9, 100 mM KCl, 1 mM DTT, 12.5 mM MgCl2, 0.05 mM EDTA, 17% glycerol) with 400 ng/μl of poly (dI-dC) (Thermo Scientific). For each binding site, a reaction was done that included an amount of GST-fusion protein ranging from 3,000 ng down to 111 ng. For each binding site, a control reaction was done that lacked GST-fusion protein. Binding reactions were carried out for 30 minutes on ice and then separated by a 5% non-denaturing polyacrylamide gel for 2 hours at 200 V. Reactions were then transferred and cross linked to a Hybond-N+ membrane (GE Healthcare Amersham) for chemiluminescent detection using the Chemiluminescent Nucleic Acid Detection Module and manufacture’s protocol (Thermo Scientific). Chemiluminescent images were taken using a BioChemi gel documentation system (UVP).

## Supporting Information

S1 Fig Mapping the CRE sequences sufficient to drive male-specific *tan* and *yellow* expression. (A) To scale representation of the *tan* locus. The t_MSE is composed of the sequence between *Gr8a* and *CG1537*, and t_MSE1-3 are three truncated forms of this larger sequence. (B-F) EGFP-reporter transgene activity driven by *tan* locus sequences in transgenic *D*. *melanogaster* male pupae at ~95 hours after puparium formation (hAPF). (B) At ~90 hours hAPF, the *D*. *melanogaster* t_MSE sequence drives EGFP reporter transgene expression throughout the A5 and A6 segments of males. In order to determine whether the t_MSE could be reduced to a smaller sequence, we created three truncated versions. Of the three truncations, the centrally positioned (D) t_MSE2 drove a pattern of expression comparable to the larger t_MSE. (C) For the t_MSE1 and (E) t_MSE3 truncations, EGFP expression was lacking at ~90 hAPF. (F) Interestingly, at ~100 hAPF the t_MSE3 fragment drove reporter expression throughout the abdomen. Overall, these results demonstrate that the key regulatory inputs for the t_MSE are localized to a 351 base pair sequence referred to as t_MSE2. (G) To scale representation of the *yellow* locus. The yBE is composed of sequence 5’ of *yellow* exon 1, which contains two binding sites for Abd-B. yBE 1.1, yBE 0.9, and yBE 0.6 are three nested versions of this larger sequence. (H-K) EGFP-reporter transgene activity driven by *yellow* locus sequences in transgenic male pupae at 85 hAPF.
(TIF)

S2 Fig Conserved synteny between *tan* and the upstream genes between which the t_MSE is located in *D*. *melanogaster*. Contiguous sequences containing the orthologous *tan* locus were identified from *Sophophora* species with sequenced genomes. A histogram plot of sequence conservation greater than 50% between the *tan* exon 8 region and the first exon of *Gr8a*. Conserved putatively non-exon sequences are shaded in salmon color, whereas conserved exon non-coding and coding sequences are shaded in teal and lavender color respectively. The location of the scanning mutant 5 region of the t_MSE is annotated between *CG1537* and *Gr8a*. Though the t_MSE sequence is not deeply conserved in this comparison, synteny between *tan*, *CG1537*, and *Gr8a* was conserved since the most recent common ancestor of *D*. *melanogaster* and *D*. *willistoni*.
(TIF)

S3 Fig The regulatory activity of the *D*. *pseudoobscura* sequence 5’ of the *yellow* gene. The abdomens of (A) male and (B) female *D*. *pseudoobscura* have a dark brown coloration. EGFP-reporter transgene activity driven by the *D*. *pseudoobscura yellow* 5’ sequence in transgenic (C) male and (D) female pupae at ~85 hours after puparium formation. The level of EGFP expression is notably higher in male abdomens compared to those for females.
(TIF)

S4 Fig Mapping the CRE architecture of the 5’ region of the *D*. *willistoni yellow* gene. (A) Two ~3kb partially overlapping sequences 5’ of the *D*. *willistoni yellow* first exon were included into EGFP-reporter transgenes. These sequences collectively cover the ~5.5 kb of genomic sequence immediately 5’ of *yellow* exon 1. Pattern of EGFP expression in the (B and C) abdomens and (B’ and C’) wings of transgenic *D*. *melanogaster* pupae at ~85 hours after puparium formation. The y wil 5’1 sequence has a regulatory activity that drives a stripe pattern on the posterior regions of each abdomen segment, a pattern which is coincident with the pigmentation pattern on *D*. *willistoni* tergites. (B’) This sequence also possesses strong CRE activity in the wing. (C and C’) The y wil 5’2 sequence lacked any noteworthy regulatory activities in the abdomen and wing.
(TIF)

S5 Fig Sequence alignment of yBE0.6 with scanning mutant versions. Blue background indicates the AscI (GGCGCGCC) and SbfI (CCTGCAGG) restriction enzymes sites that were added for cloning into the reporter transgene vector. Gray background and black letters indicates sequences that comprise a scanning mutation of non-complementary transversions and for which there was not a resulting alteration in the male abdomen regulatory activity. Red background and black letters indicates sequences that comprise a scanning mutation of non-complementary transversions and in which the mutant CRE had a reduced regulatory activity in the male abdomen. Blue background and white letters indicates sequences that comprise a scanning mutation of non-complementary transversions and for which the mutant CRE had an increased regulatory activity in the male abdomen. The lower case nucleotide letters indicate the non-complementary transversions. The black background with white letters indicates the Abd-B sites indentified in Jeong et al., 2006; which were not mutated in this study.
(DOC)

S6 Fig Mapping functional *yellow* regulatory sequences through CRE scanning mutagenesis. (A) Name and location of yBE0.6 scanning mutations and the wild type pattern of EGFP expression in transgenic *D*. *melanogaster* pupae. Scanning mutations are indicated as red blocks and vertical blue lines indicate the position of two Abd-B binding sites that were not mutated in this analysis. (B-K) The EGFP expression pattern in the male abdomen at ~85 hours after puparium formation driven by yBE0.6 scan mutant sequences.
(TIF)

S7 Fig Sequence alignment of t_MSE with scanning mutant versions. Blue background indicates the AscI (GGCGCGCC) and SbfI (CCTGCAGG) restriction enzymes sites that were added for cloning into the reporter transgene vector. Gray background and black letters indicates sequences that comprise a scanning mutation of non-complementary transversions and for which there was not a resulting alteration in the male abdomen regulatory activity. Red background and black letters indicate sequences that comprise a scanning mutation of non-complementary transversions and for which the mutant CRE had a reduced regulatory activity in the male abdomen. The lower case nucleotide letters indicate the non-complementary transversions.
(DOC)

S8 Fig Mapping functional *tan* regulatory sequences through CRE scanning mutagenesis. (A) Name and location of t_MSE scanning mutations and the wild type pattern of EGFP expression in transgenic *D*. *melanogaster* pupae. Scanning mutations are indicated as red blocks and the region between the dashed vertical blue lines indicates the sequence considered to be the t_MSE2. (B-K) EGFP expression pattern at ~95 hours after puparium formation driven by t_MSE scanning mutant sequences in male abdomens.
(TIF)

S9 Fig Sequence alignment of t_MSE2 with scanning mutant versions. Blue background indicates the AscI (GGCGCGCC) and SbfI (CCTGCAGG) restriction enzymes sites that were added for cloning into the reporter transgene vector. Gray background and black letters indicates sequences that comprise a scanning mutation of non-complementary transversions and for which there was not a resulting alteration in the male abdomen regulatory activity. Red background and black letters indicates sequences that comprise a scanning mutation of non-complementary transversions and for which the mutant CRE had a reduced regulatory activity in the male abdomen. The region with a green background and white letters indicates the region where the SM5 mutation was originally made. The lower case nucleotide letters indicate the non-complementary transversions.
(DOC)

S10 Fig Fine-scale mapping of functional *tan* regulatory sequences through CRE scanning mutagenesis. (A) Name and location of t_MSE2 scanning mutations and the wild type pattern of EGFP expression in transgenic *D*. *melanogaster* pupae. Numbered blue blocks indicate the position of putative Hox-sites in the scanning mutant 6 region. Region between the vertical dashed red lines is the SM5 and SM6 regions that were identified as being necessary for t_MSE activity. The locations of scanning mutations in SM 5i1-SM 6i6 are indicated as red regions. The red boxes indicate the putative Hox-sites that were mutated in the t_MSE2 TTAT KO and TTAT+TAAT KO sequences. (B-O) EGFP expression pattern at ~95 hours after puparium formation driven by t_MSE2 mutant sequences in male abdomens. Compared to the regulatory of the wild type t_MSE2, the activity of the t_MSE2 SM5i1 sequence is 114±3% in the A5 segment.
(TIF)

S11 Fig Sequence alignment of t_MSE2 with Hox site mutant versions. Blue background indicates the AscI (GGCGCGCC) and SbfI (CCTGCAGG) restriction enzymes sites that were added for cloning into the reporter transgene vector. Black background indicates Hox site sequences that were mutated, with the substituted nucleotides shown in lowercase red letters. Teal background indicates the Hth site that was mutated, with the substituted nucleotides shown in white letters.
(DOC)

S12 Fig *In vitro* interactions between the DNA binding domains of Hox proteins and a known Hox site. (A-C) Gel shift assays between annealed oligonucleotide probes for a *Dll* CRE sequence and the GST-Abd-A DNA binding domain fusion protein and the GST-Abd-B DNA binding domain fusion protein. A mutant version of the probe was tested that possessed a mutation in the known Abd-A binding site. Binding reactions used increasing amounts of the GST-DNA binding domain fusion protein (from left to right: 0 ng, 111 ng, 333 ng, 1000 ng, and 3000 ng).
(TIF)

S1 Table Primers used to make *in situ* hybridization probes. (DOCX)

S2 Table Primers used to clone *D*. *melanogaster yellow* and *tan* gene sequences. (DOCX)

S3 Table Primers used to clone orthologous *yellow* and *tan* loci sequences. (DOCX)

S4 Table Oligonucleotide sequences used to make gel shift assay binding sites. (DOCX)
