# Supplementary figures and images for: The Evolutionary Origination and Diversification of a Dimorphic Gene Regulatory Network through Parallel Innovations in cis and trans
Source: PLoS Genet. 2015 Apr 2;11(4):e1005136. doi: 10.1371/journal.pgen.1005136 (PMC4383587; doi:10.1371/journal.pgen.1005136)

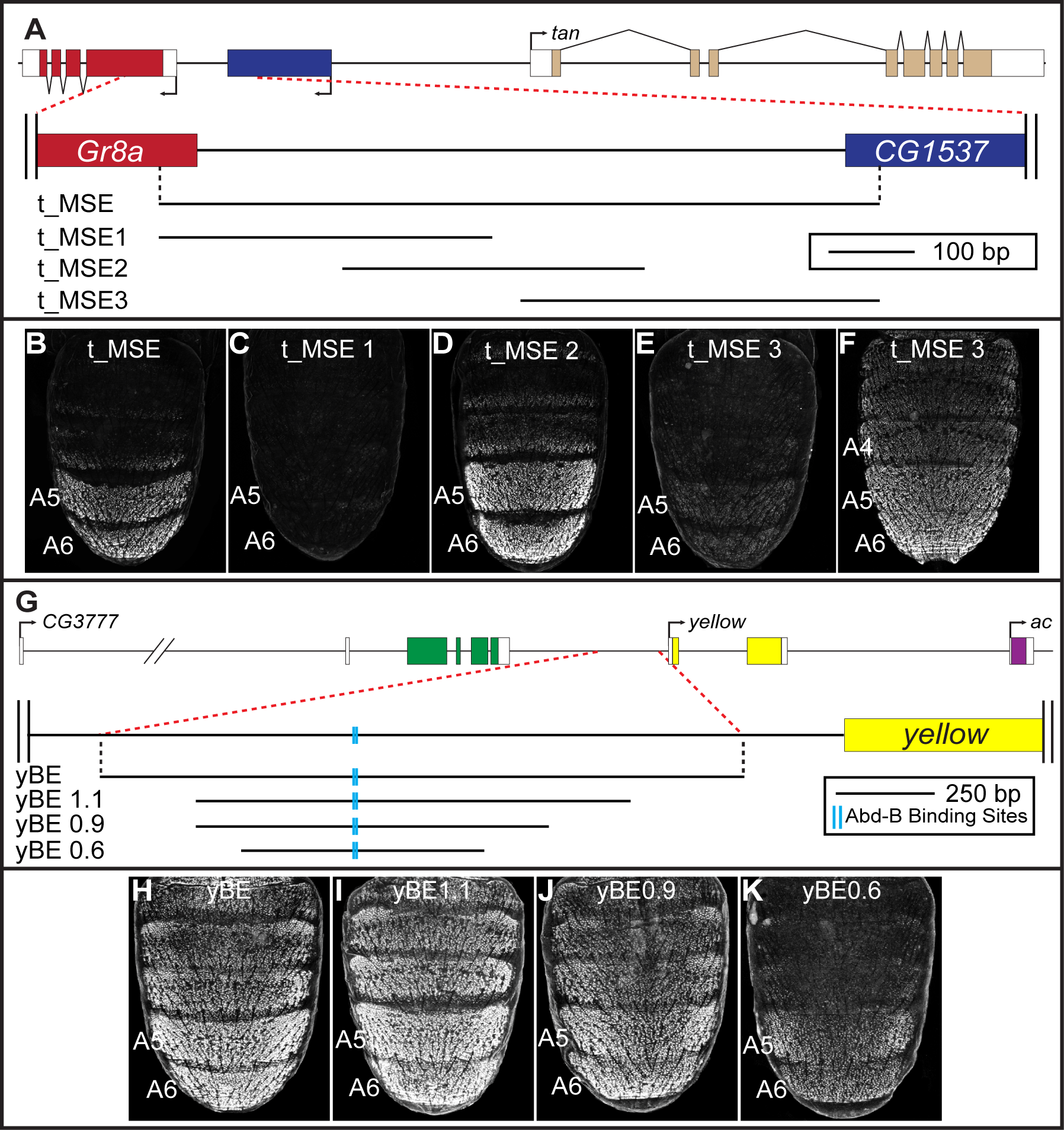

Supplement: S1 Fig — (A) To scale representation of the tan locus. The t_MSE is composed of the sequence between Gr8a and CG1537, and t_MSE1-3 are three truncated forms of this larger sequence. (B-F) EGFP-reporter transgene activity driven by tan locus sequences in transgenic D. melanogaster male pupae at ~95 hours after puparium formation (hAPF). (B) At ~90 hours hAPF, the D. melanogaster t_MSE sequence drives EGFP reporter transgene expression throughout the A5 and A6 segments of males. In order to determine whether the t_MSE could be reduced to a smaller sequence, we created three truncated versions. Of the three truncations, the centrally positioned (D) t_MSE2 drove a pattern of expression comparable to the larger t_MSE. (C) For the t_MSE1 and (E) t_MSE3 truncations, EGFP expression was lacking at ~90 hAPF. (F) Interestingly, at ~100 hAPF the t_MSE3 fragment drove reporter expression throughout the abdomen. Overall, these results demonstrate that the key regulatory inputs for the t_MSE are localized to a 351 base pair sequence referred to as t_MSE2. (G) To scale representation of the yellow locus. The yBE is composed of sequence 5’ of yellow exon 1, which contains two binding sites for Abd-B. yBE 1.1, yBE 0.9, and yBE 0.6 are three nested versions of this larger sequence. (H-K) EGFP-reporter transgene activity driven by yellow locus sequences in transgenic male pupae at 85 hAPF. (TIF) [file pgen.1005136.s001.tif]

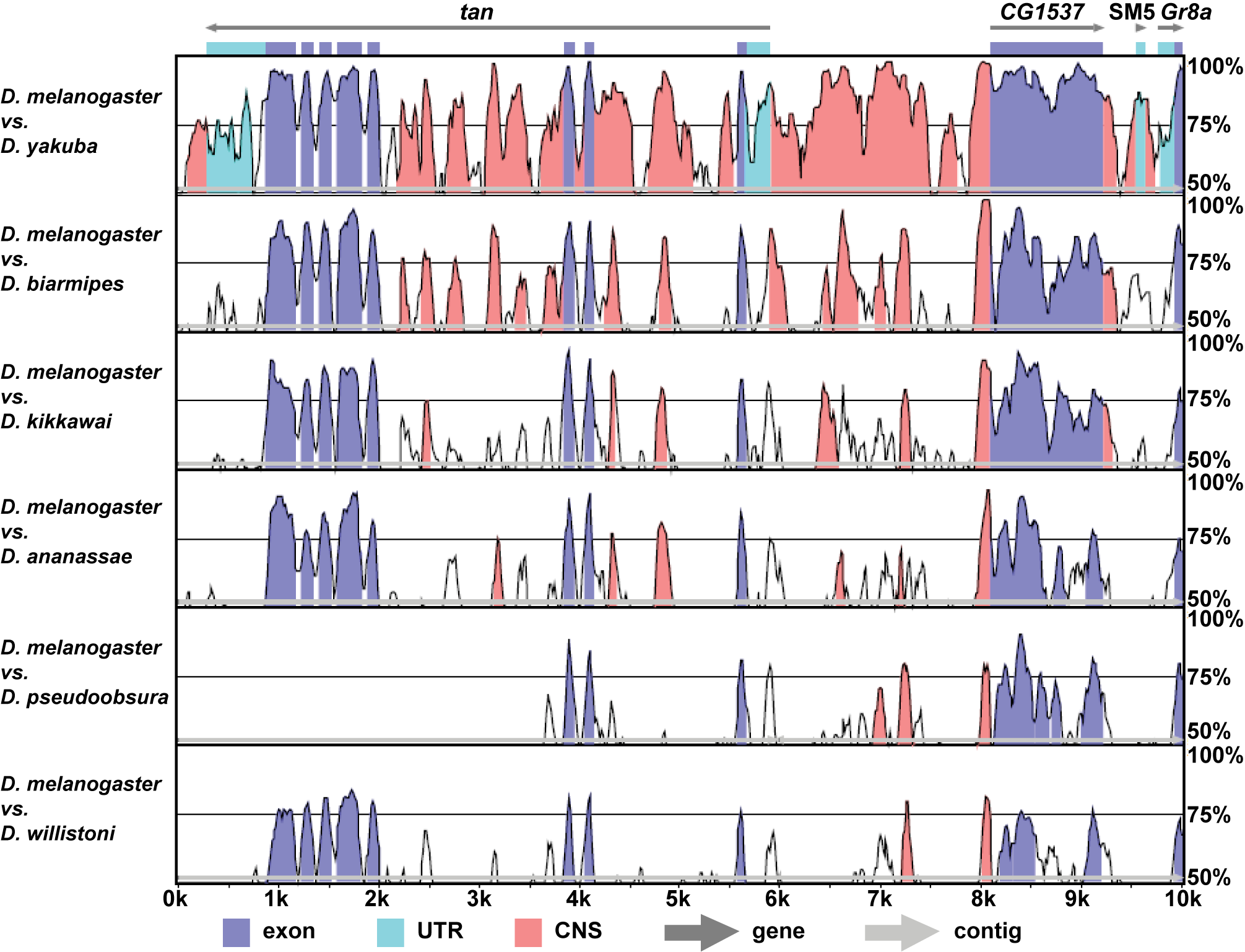

Supplement: S2 Fig — Contiguous sequences containing the orthologous tan locus were identified from Sophophora species with sequenced genomes. A histogram plot of sequence conservation greater than 50% between the tan exon 8 region and the first exon of Gr8a. Conserved putatively non-exon sequences are shaded in salmon color, whereas conserved exon non-coding and coding sequences are shaded in teal and lavender color respectively. The location of the scanning mutant 5 region of the t_MSE is annotated between CG1537 and Gr8a. Though the t_MSE sequence is not deeply conserved in this comparison, synteny between tan, CG1537, and Gr8a was conserved since the most recent common ancestor of D. melanogaster and D. willistoni. (TIF) [file pgen.1005136.s002.tif]

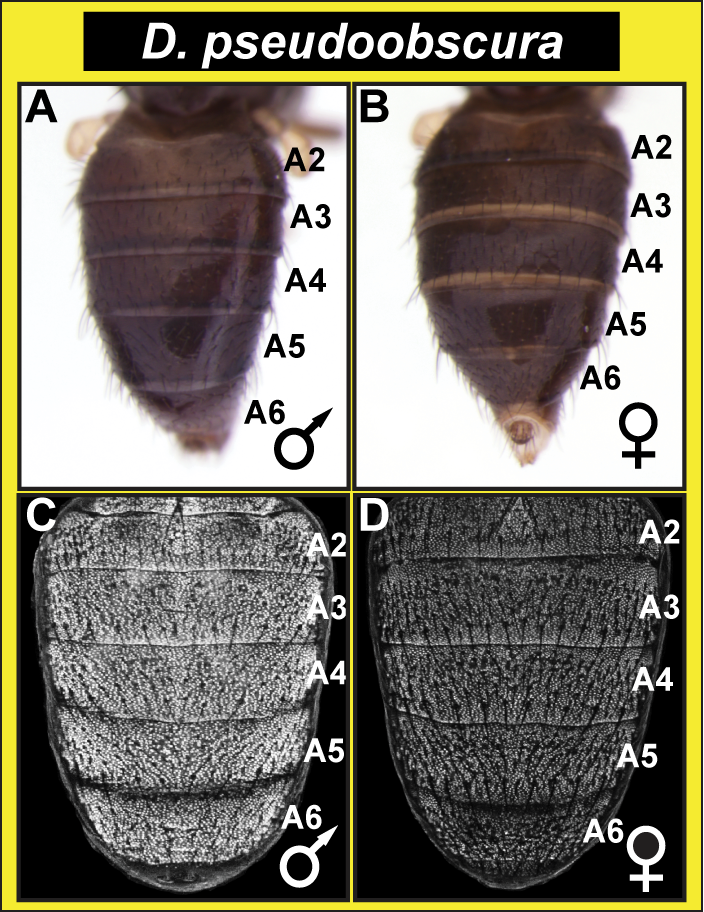

Supplement: S3 Fig — The abdomens of (A) male and (B) female D. pseudoobscura have a dark brown coloration. EGFP-reporter transgene activity driven by the D. pseudoobscura yellow 5’ sequence in transgenic (C) male and (D) female pupae at ~85 hours after puparium formation. The level of EGFP expression is notably higher in male abdomens compared to those for females. (TIF) [file pgen.1005136.s003.tif]

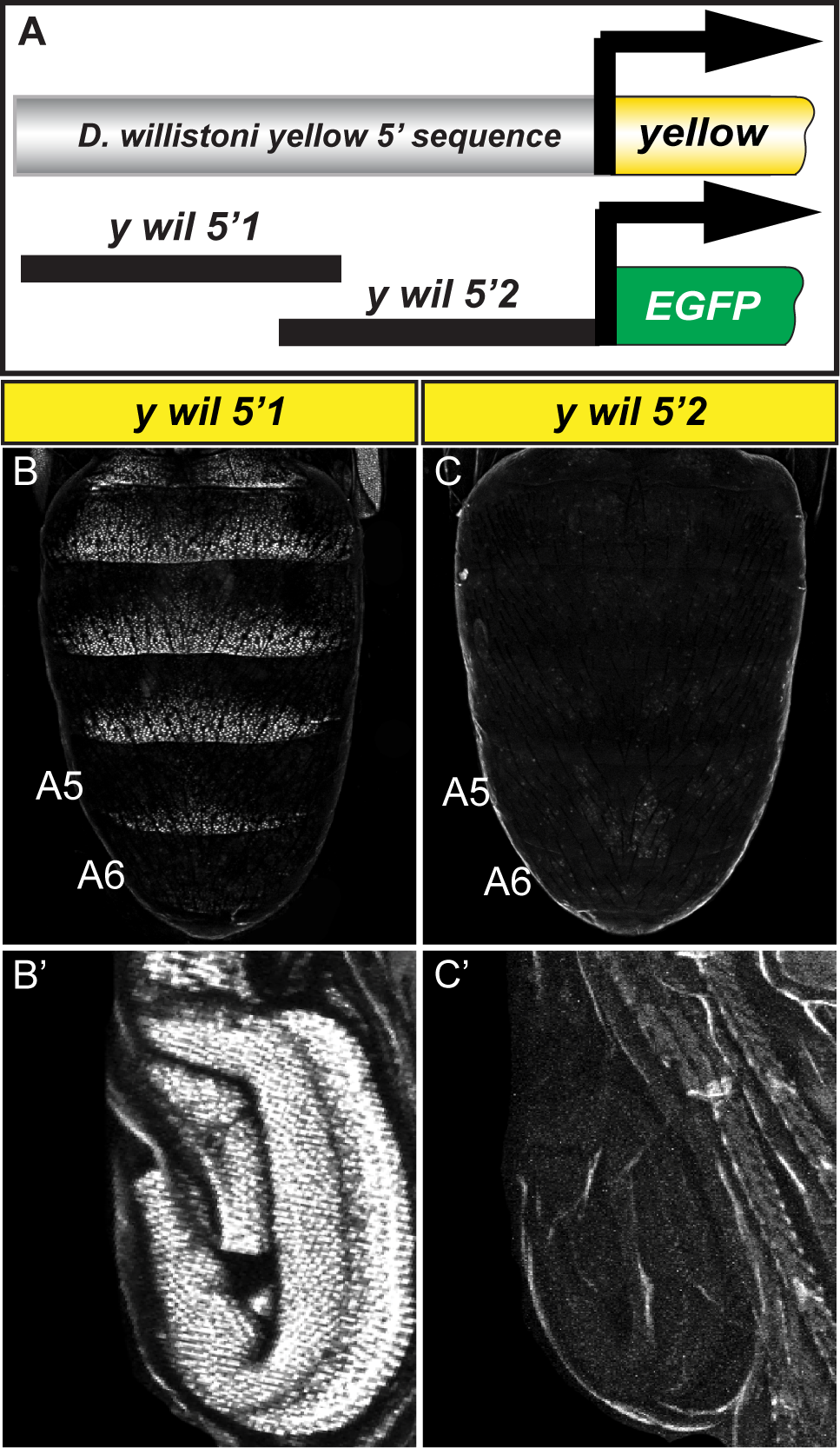

Supplement: S4 Fig — (A) Two ~3kb partially overlapping sequences 5’ of the D. willistoni yellow first exon were included into EGFP-reporter transgenes. These sequences collectively cover the ~5.5 kb of genomic sequence immediately 5’ of yellow exon 1. Pattern of EGFP expression in the (B and C) abdomens and (B’ and C’) wings of transgenic D. melanogaster pupae at ~85 hours after puparium formation. The y wil 5’1 sequence has a regulatory activity that drives a stripe pattern on the posterior regions of each abdomen segment, a pattern which is coincident with the pigmentation pattern on D. willistoni tergites. (B’) This sequence also possesses strong CRE activity in the wing. (C and C’) The y wil 5’2 sequence lacked any noteworthy regulatory activities in the abdomen and wing. (TIF) [file pgen.1005136.s004.tif]

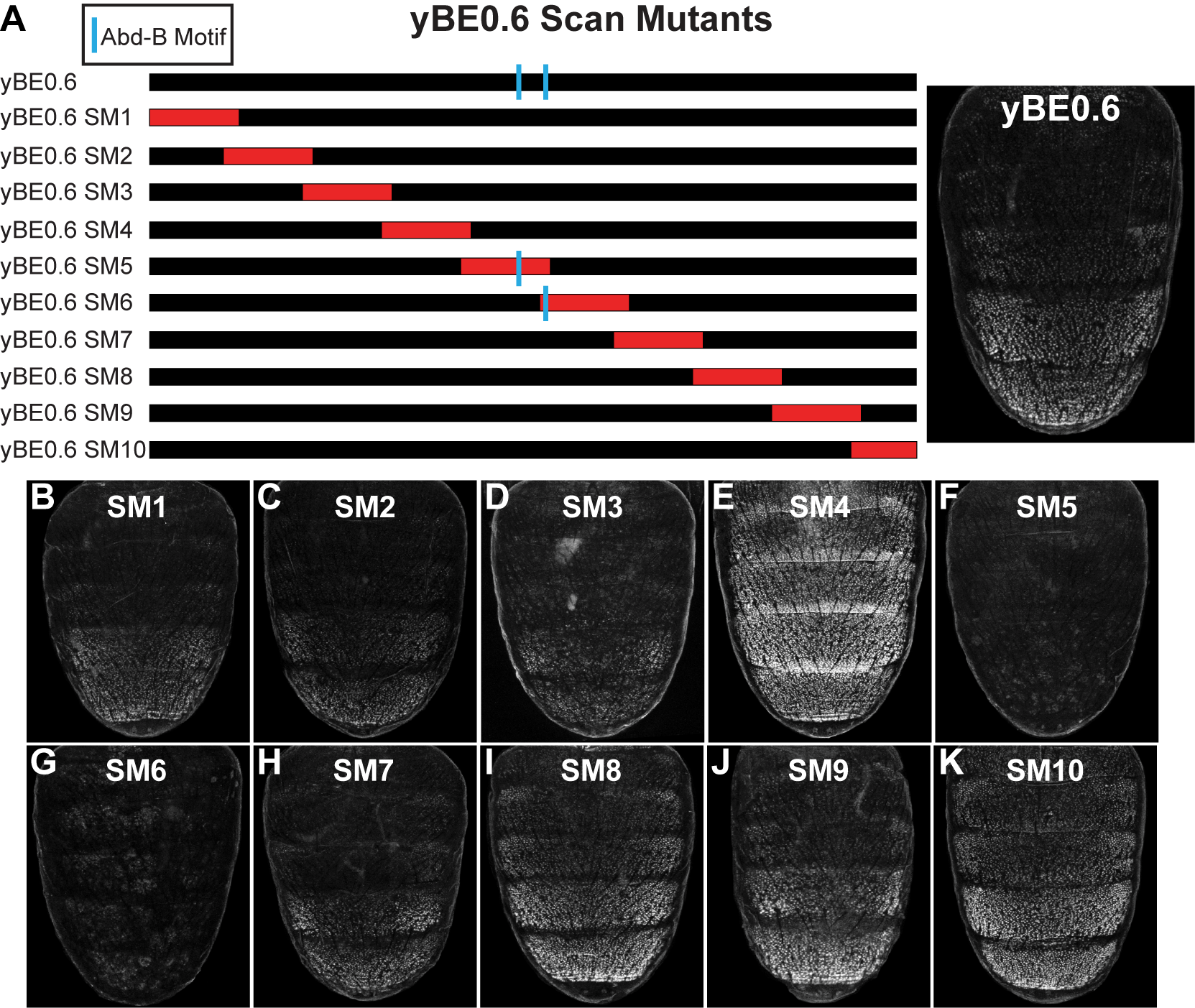

Supplement: S6 Fig — (A) Name and location of yBE0.6 scanning mutations and the wild type pattern of EGFP expression in transgenic D. melanogaster pupae. Scanning mutations are indicated as red blocks and vertical blue lines indicate the position of two Abd-B binding sites that were not mutated in this analysis. (B-K) The EGFP expression pattern in the male abdomen at ~85 hours after puparium formation driven by yBE0.6 scan mutant sequences. (TIF) [file pgen.1005136.s006.tif]

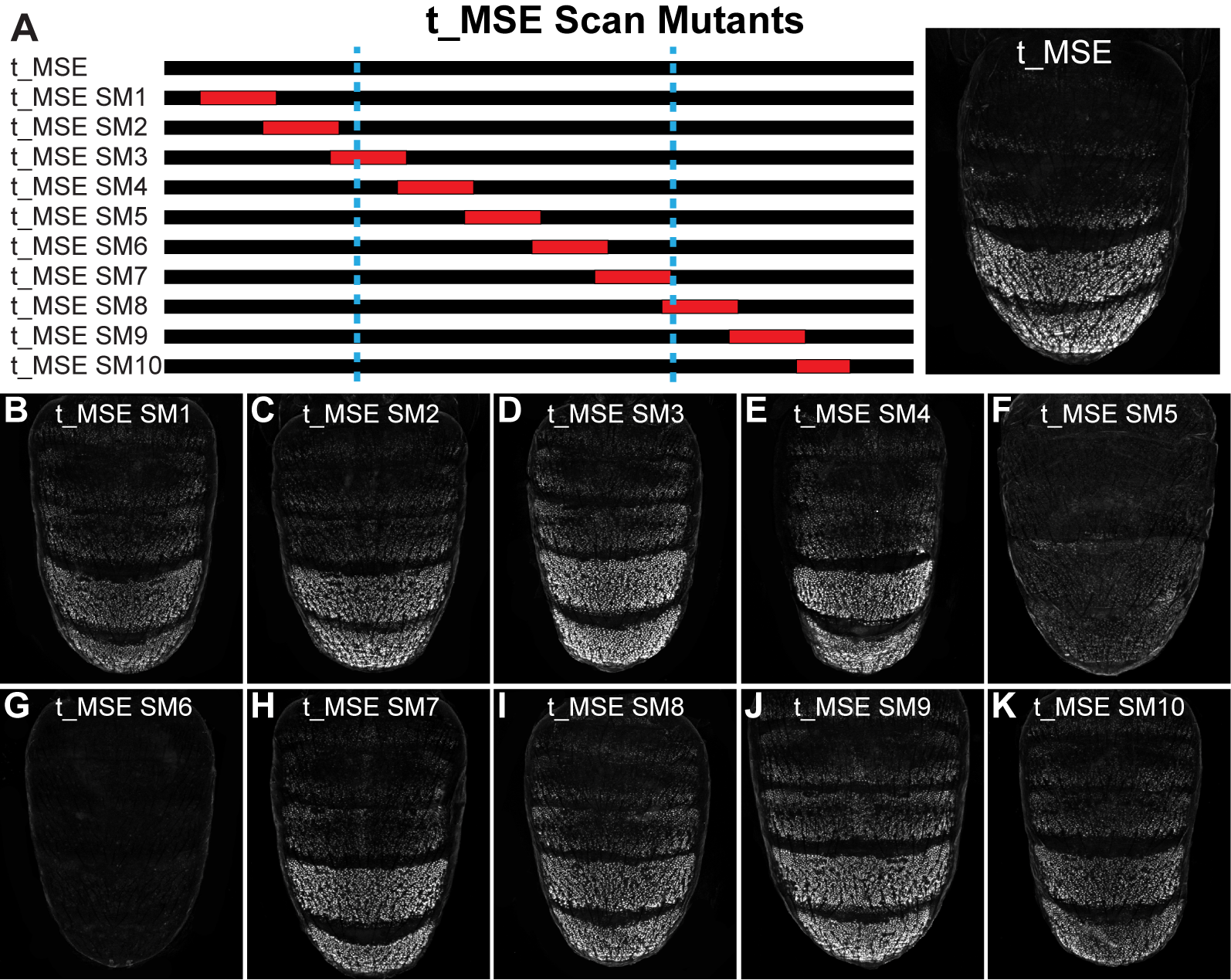

Supplement: S8 Fig — (A) Name and location of t_MSE scanning mutations and the wild type pattern of EGFP expression in transgenic D. melanogaster pupae. Scanning mutations are indicated as red blocks and the region between the dashed vertical blue lines indicates the sequence considered to be the t_MSE2. (B-K) EGFP expression pattern at ~95 hours after puparium formation driven by t_MSE scanning mutant sequences in male abdomens. (TIF) [file pgen.1005136.s008.tif]

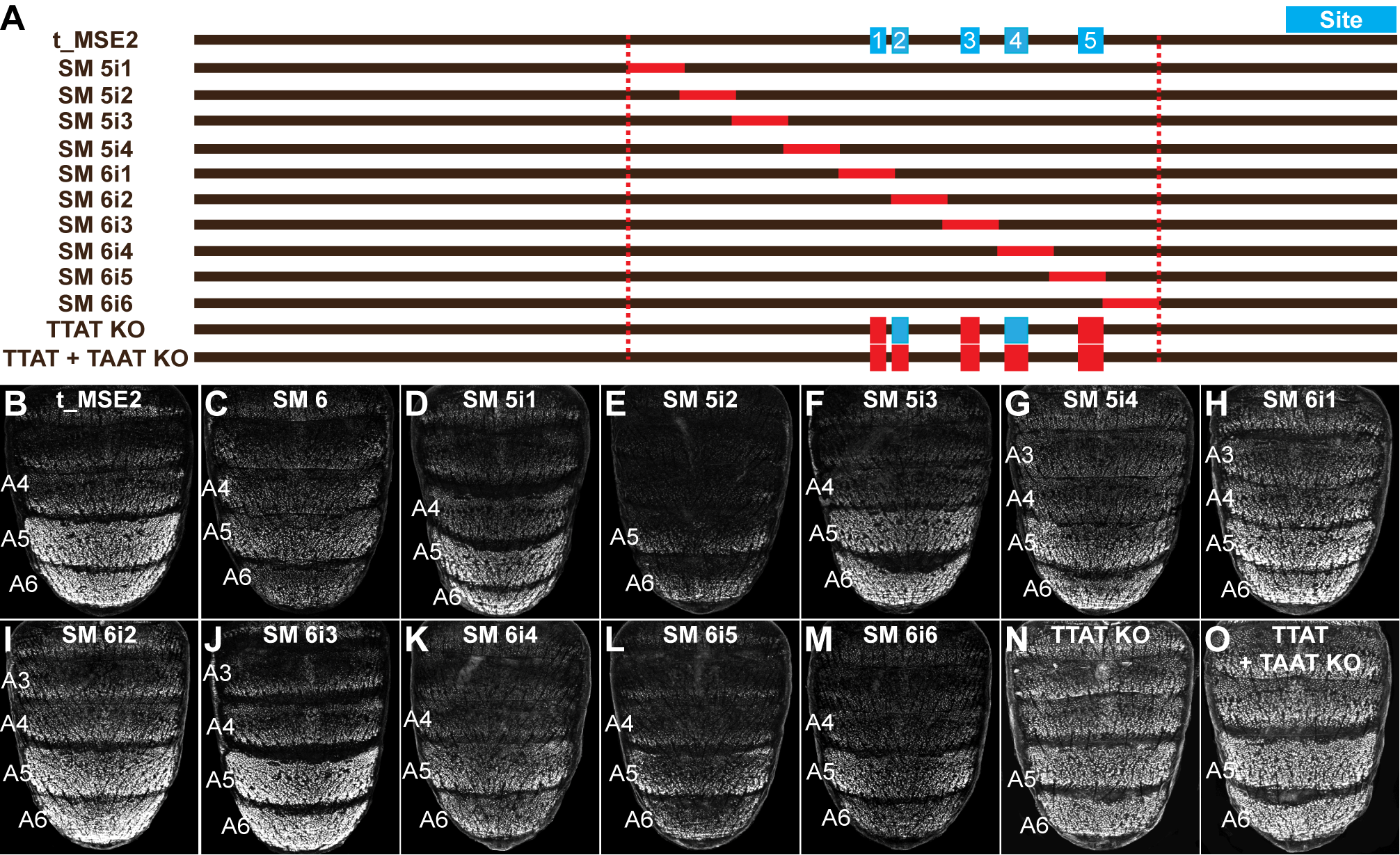

Supplement: S10 Fig — (A) Name and location of t_MSE2 scanning mutations and the wild type pattern of EGFP expression in transgenic D. melanogaster pupae. Numbered blue blocks indicate the position of putative Hox-sites in the scanning mutant 6 region. Region between the vertical dashed red lines is the SM5 and SM6 regions that were identified as being necessary for t_MSE activity. The locations of scanning mutations in SM 5i1-SM 6i6 are indicated as red regions. The red boxes indicate the putative Hox-sites that were mutated in the t_MSE2 TTAT KO and TTAT+TAAT KO sequences. (B-O) EGFP expression pattern at ~95 hours after puparium formation driven by t_MSE2 mutant sequences in male abdomens. Compared to the regulatory of the wild type t_MSE2, the activity of the t_MSE2 SM5i1 sequence is 114±3% in the A5 segment. (TIF) [file pgen.1005136.s010.tif]

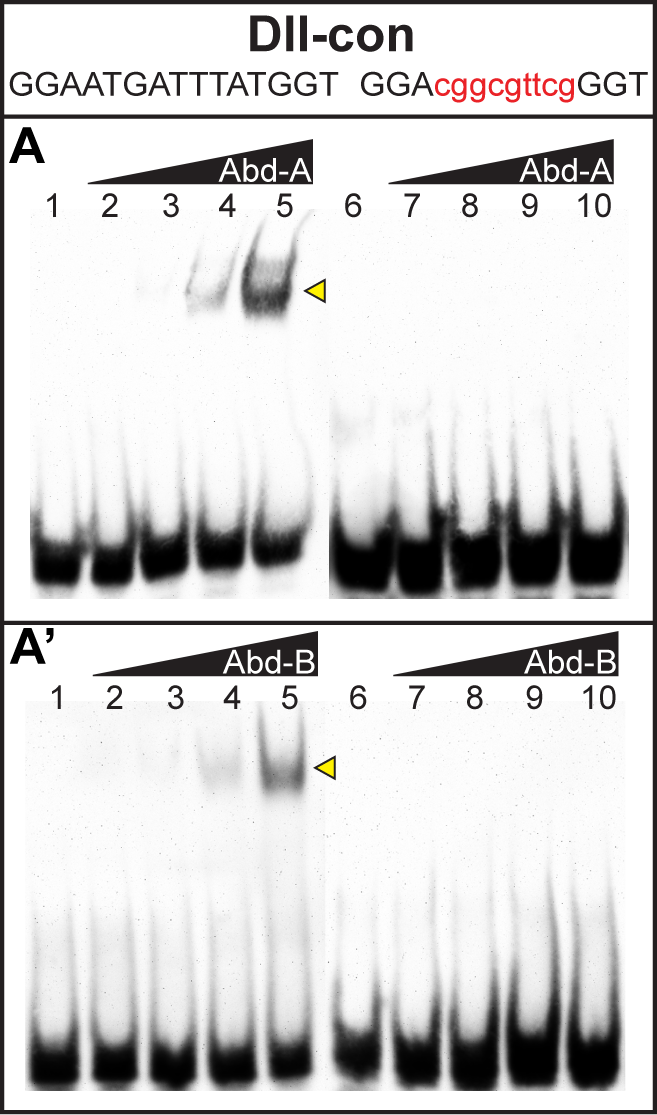

Supplement: S12 Fig — (A-C) Gel shift assays between annealed oligonucleotide probes for a Dll CRE sequence and the GST-Abd-A DNA binding domain fusion protein and the GST-Abd-B DNA binding domain fusion protein. A mutant version of the probe was tested that possessed a mutation in the known Abd-A binding site. Binding reactions used increasing amounts of the GST-DNA binding domain fusion protein (from left to right: 0 ng, 111 ng, 333 ng, 1000 ng, and 3000 ng). (TIF) [file pgen.1005136.s012.tif]
