# Supplementary material for: The Evolutionary Origination and Diversification of a Dimorphic Gene Regulatory Network through Parallel Innovations in cis and trans
Source: PLoS Genet. 2015 Apr 2;11(4):e1005136. doi: 10.1371/journal.pgen.1005136 (PMC4383587; doi:10.1371/journal.pgen.1005136)
Supplement: S7 Fig — Blue background indicates the AscI (GGCGCGCC) and SbfI (CCTGCAGG) restriction enzymes sites that were added for cloning into the reporter transgene vector. Gray background and black letters indicates sequences that comprise a scanning mutation of non-complementary transversions and for which there was not a resulting alteration in the male abdomen regulatory activity. Red background and black letters indicate sequences that comprise a scanning mutation of non-complementary transversions and for which the mutant CRE had a reduced regulatory activity in the male abdomen. The lower case nucleotide letters indicate the non-complementary transversions. (DOC) [file pgen.1005136.s007.doc]

**AscI**

t_MSE 1 GGCGCGCCCC ATGGAAGCCG AGCACCTGGT AGAGCCGCAG GTGGAACTGC

t_MSE SM1 1 GGCGCGCCCC ATGGAAGCCG AGCACCTGGT AGAGCCGCAG GTGGAACTGC

t_MSE SM2 1 GGCGCGCCCC ATGGAAGCCG AGCACCTGGT AGAGCCGCAG GTGGAACTGC

t_MSE SM3 1 GGCGCGCCCC ATGGAAGCCG AGCACCTGGT AGAGCCGCAG GTGGAACTGC

t_MSE SM4 1 GGCGCGCCCC ATGGAAGCCG AGCACCTGGT AGAGCCGCAG GTGGAACTGC

t_MSE SM5 1 GGCGCGCCCC ATGGAAGCCG AGCACCTGGT AGAGCCGCAG GTGGAACTGC

t_MSE SM6 1 GGCGCGCCCC ATGGAAGCCG AGCACCTGGT AGAGCCGCAG GTGGAACTGC

t_MSE SM7 1 GGCGCGCCCC ATGGAAGCCG AGCACCTGGT AGAGCCGCAG GTGGAACTGC

t_MSE SM8 1 GGCGCGCCCC ATGGAAGCCG AGCACCTGGT AGAGCCGCAG GTGGAACTGC

t_MSE SM9 1 GGCGCGCCCC ATGGAAGCCG AGCACCTGGT AGAGCCGCAG GTGGAACTGC

t_MSE SM10 1 GGCGCGCCCC ATGGAAGCCG AGCACCTGGT AGAGCCGCAG GTGGAACTGC

t_MSE 43 AGGACCCGAC CCAGATGGCC GCTCATCGTT GACTGCTCGG AAGTGAAACC

t_MSE SM1 51 AGGACCCGAC CCAGATGGCC GCTCATaGgT tAaTtCgCtG cAtTtAcAaC

t_MSE SM2 51 AGGACCCGAC CCAGATGGCC GCTCATCGTT GACTGCTCGG AAGTGAAACC

t_MSE SM3 51 AGGACCCGAC CCAGATGGCC GCTCATCGTT GACTGCTCGG AAGTGAAACC

t_MSE SM4 51 AGGACCCGAC CCAGATGGCC GCTCATCGTT GACTGCTCGG AAGTGAAACC

t_MSE SM5 51 AGGACCCGAC CCAGATGGCC GCTCATCGTT GACTGCTCGG AAGTGAAACC

t_MSE SM6 51 AGGACCCGAC CCAGATGGCC GCTCATCGTT GACTGCTCGG AAGTGAAACC

t_MSE SM7 51 AGGACCCGAC CCAGATGGCC GCTCATCGTT GACTGCTCGG AAGTGAAACC

t_MSE SM8 51 AGGACCCGAC CCAGATGGCC GCTCATCGTT GACTGCTCGG AAGTGAAACC

t_MSE SM9 51 AGGACCCGAC CCAGATGGCC GCTCATCGTT GACTGCTCGG AAGTGAAACC

t_MSE SM10 51 AGGACCCGAC CCAGATGGCC GCTCATCGTT GACTGCTCGG AAGTGAAACC

t_MSE 93 CTTATGGATG GACAGGCCTT ATCCTTGCGG CGGATCTCCC TTTAAATGGG

t_MSE SM1 101 aTgAgGtAgG tAaAtGaCgT cTaCgTtCtG aGtAgCgCaC gTgAcAgGtG

t_MSE SM2 101 CTTATGGATG GACAGGCCTT ATCCTTGCGG CGGATCTCCC TTTAAATGGG

t_MSE SM3 101 CTTATGGATG GACAGGCCTT ATCCTTGCGG CGGATCTCCC TTTAAATGGG

t_MSE SM4 101 CTTATGGATG GACAGGCCTT ATCCTTGCGG CGGATCTCCC TTTAAATGGG

t_MSE SM5 101 CTTATGGATG GACAGGCCTT ATCCTTGCGG CGGATCTCCC TTTAAATGGG

t_MSE SM6 101 CTTATGGATG GACAGGCCTT ATCCTTGCGG CGGATCTCCC TTTAAATGGG

t_MSE SM7 101 CTTATGGATG GACAGGCCTT ATCCTTGCGG CGGATCTCCC TTTAAATGGG

t_MSE SM8 101 CTTATGGATG GACAGGCCTT ATCCTTGCGG CGGATCTCCC TTTAAATGGG

t_MSE SM9 101 CTTATGGATG GACAGGCCTT ATCCTTGCGG CGGATCTCCC TTTAAATGGG

t_MSE SM10 101 CTTATGGATG GACAGGCCTT ATCCTTGCGG CGGATCTCCC TTTAAATGGG

t_MSE 143 CCAACAAACA ACTGAGAAAT CCATTAGCCT AACTGACTTC CTCAAAAACA

t_MSE SM1 151 aCcAaAcAaA cCTGAGAAAT CCATTAGCCT AACTGACTTC CTCAAAAACA

t_MSE SM2 151 CaccaAAcaA cCgGcGcAcT aCcTgAtCaT cAaTtAaTgC aTaAcAcAaA

t_MSE SM3 151 CCAACAAACA ACTGAGAAAT CCATTAGCCT AACTGACTTC CTCAAAAACA

t_MSE SM4 151 CCAACAAACA ACTGAGAAAT CCATTAGCCT AACTGACTTC CTCAAAAACA

t_MSE SM5 151 CCAACAAACA ACTGAGAAAT CCATTAGCCT AACTGACTTC CTCAAAAACA

t_MSE SM6 151 CCAACAAACA ACTGAGAAAT CCATTAGCCT AACTGACTTC CTCAAAAACA

t_MSE SM7 151 CCAACAAACA ACTGAGAAAT CCATTAGCCT AACTGACTTC CTCAAAAACA

t_MSE SM8 151 CCAACAAACA ACTGAGAAAT CCATTAGCCT AACTGACTTC CTCAAAAACA

t_MSE SM9 151 CCAACAAACA ACTGAGAAAT CCATTAGCCT AACTGACTTC CTCAAAAACA

t_MSE SM10 151 CCAACAAACA ACTGAGAAAT CCATTAGCCT AACTGACTTC CTCAAAAACA

t_MSE 193 CTGAGAACAG TAGTCCATGT ATGAATGTAC CAAACACGAT TTCCGTATTT

t_MSE SM1 201 CTGAGAACAG TAGTCCATGT ATGAATGTAC CAAACACGAT TTCCGTATTT

t_MSE SM2 201 aTtAtAcCcG gAtTaCcTtT cTtAcTtTcC aAcAaACGAT TTCCGTATTT

t_MSE SM3 201 CTGAGAACAG TAGTCCATGT ATGAcTtTcC aAcAaAaGcT gTaCtTcTgT

t_MSE SM4 201 CTGAGAACAG TAGTCCATGT ATGAATGTAC CAAACACGAT TTCCGTATTT

t_MSE SM5 201 CTGAGAACAG TAGTCCATGT ATGAATGTAC CAAACACGAT TTCCGTATTT

t_MSE SM6 201 CTGAGAACAG TAGTCCATGT ATGAATGTAC CAAACACGAT TTCCGTATTT

t_MSE SM7 201 CTGAGAACAG TAGTCCATGT ATGAATGTAC CAAACACGAT TTCCGTATTT

t_MSE SM8 201 CTGAGAACAG TAGTCCATGT ATGAATGTAC CAAACACGAT TTCCGTATTT

t_MSE SM9 201 CTGAGAACAG TAGTCCATGT ATGAATGTAC CAAACACGAT TTCCGTATTT

t_MSE SM10 201 CTGAGAACAG TAGTCCATGT ATGAATGTAC CAAACACGAT TTCCGTATTT

t_MSE 243 GAAATAATAA TAAATAATCA GAATGTAAAT ATATTATACG TTTTATAGAT

t_MSE SM1 251 GAAATAATAA TAAATAATCA GAATGTAAAT ATATTATACG TTTTATAGAT

t_MSE SM2 251 GAAATAATAA TAAATAATCA GAATGTAAAT ATATTATACG TTTTATAGAT

t_MSE SM3 251 tAcAgAcTcA gAcAgAcTaA tAcTtTcAcT cTcTgAgAaG gTgTcTcGcT

t_MSE SM4 251 GAAATAATAA TAAATAATCA GAATGTAAAT ATATTATACG TTTTATAGCT

t_MSE SM5 251 GAAATAATAA TAAATAATCA GAATGTAAAT ATATTATACG TTTTATAGAT

t_MSE SM6 251 GAAATAATAA TAAATAATCA GAATGTAAAT ATATTATACG TTTTATAGAT

t_MSE SM7 251 GAAATAATAA TAAATAATCA GAATGTAAAT ATATTATACG TTTTATAGAT

t_MSE SM8 251 GAAATAATAA TAAATAATCA GAATGTAAAT ATATTATACG TTTTATAGAT

t_MSE SM9 251 GAAATAATAA TAAATAATCA GAATGTAAAT ATATTATACG TTTTATAGAT

t_MSE SM10 251 GAAATAATAA TAAATAATCA GAATGTAAAT ATATTATACG TTTTATAGAT

t_MSE 293 AGAATCAAGA CTTAGGATAA TTGCACTAAG TAGTATACTT AAATTCCCAT

t_MSE SM1 301 AGAATCAAGA CTTAGGATAA TTGCACTAAG TAGTATACTT AAATTCCCAT

t_MSE SM2 301 AGAATCAAGA CTTAGGATAA TTGCACTAAG TAGTATACTT AAATTCCCAT

t_MSE SM3 301 cGcAgCaAtA CTTAGGATAA TTGCACTAAG TAGTATACTT AAATTCCCAT

t_MSE SM4 301 cGcAgCcAtA aTgAtGcTcA gTtCcCgAcG gAtTcTcCgT cAcTgCaCcT

t_MSE SM5 301 AGAATCAAGA CTTAGGATAA TTGCACTAAG TAGTATACTT AAATTCCCAT

t_MSE SM6 301 AGAATCAAGA CTTAGGATAA TTGCACTAAG TAGTATACTT AAATTCCCAT

t_MSE SM7 301 AGAATCAAGA CTTAGGATAA TTGCACTAAG TAGTATACTT AAATTCCCAT

t_MSE SM8 301 AGAATCAAGA CTTAGGATAA TTGCACTAAG TAGTATACTT AAATTCCCAT

t_MSE SM9 301 AGAATCAAGA CTTAGGATAA TTGCACTAAG TAGTATACTT AAATTCCCAT

t_MSE SM10 301 AGAATCAAGA CTTAGGATAA TTGCACTAAG TAGTATACTT AAATTCCCAT

**SM5**

t_MSE 343 TGCCAAGTGA ACCGGTTGGT ATCCAAAGTT GAAGTCAATA ACAAAAATGA

t_MSE SM1 351 TGCCAAGTGA ACCGGTTGGT ATCCAAAGTT GAAGTCAATA ACAAAAATGA

t_MSE SM2 351 TGCCAAGTGA ACCGGTTGGT ATCCAAAGTT GAAGTCAATA ACAAAAATGA

t_MSE SM3 351 TGCCAAGTGA ACCGGTTGGT ATCCAAAGTT GAAGTCAATA ACAAAAATGA

t_MSE SM4 351 gGaCcAtTtA cCaGtTgGtT cTaCaAcGtT tAcGTCAATA ACAAAAATGA

t_MSE SM5 351 TGCCAAGTGA ACCGGTTGGT ATAcCaCgGt TacGgCcAgA cCcAcAcTtA

t_MSE SM6 351 TGCCAAGTGA ACCGGTTGGT ATCCAAAGTT GAAGTCAATA ACAAAAATGA

t_MSE SM7 351 TGCCAAGTGA ACCGGTTGGT ATCCAAAGTT GAAGTCAATA ACAAAAATGA

t_MSE SM8 351 TGCCAAGTGA ACCGGTTGGT ATCCAAAGTT GAAGTCAATA ACAAAAATGA

t_MSE SM9 351 TGCCAAGTGA ACCGGTTGGT ATCCAAAGTT GAAGTCAATA ACAAAAATGA

t_MSE SM10 351 TGCCAAGTGA ACCGGTTGGT ATCCAAAGTT GAAGTCAATA ACAAAAATGA

**SM5**

**SM6**

t_MSE 393 GTGCATTTTA CTCTTGCACC ATTAGAATAT TAGATTTTAG TGTTTAAATA

t_MSE SM1 401 GTGCATTTTA CTCTTGCACC ATTAGAATAT TAGATTTTAG TGTTTAAATA

t_MSE SM2 401 GTGCATTTTA CTCTTGCACC ATTAGAATAT TAGATTTTAG TGTTTAAATA

t_MSE SM3 401 GTGCATTTTA CTCTTGCACC ATTAGAATAT TAGATTTTAG TGTTTAAATA

t_MSE SM4 401 GTGCATTTTA CTCTTGCACC ATTAGAATAT TAGATTTTAG TGTTTAAATA

t_MSE SM5 401 tTtCcTgTgA aTaTgGaAaC cTgAtAcTcT gAtAgTgTcG gGgTgAcAgA

t_MSE SM6 401 GTGCATTTTA CTCTTGCACC ATTAGAATAT TAGATTTTAG TGTTTAcAgA

t_MSE SM7 401 GTGCATTTTA CTCTTGCACC ATTAGAATAT TAGATTTTAG TGTTTAAATA

t_MSE SM8 401 GTGCATTTTA CTCTTGCACC ATTAGAATAT TAGATTTTAG TGTTTAAATA

t_MSE SM9 401 GTGCATTTTA CTCTTGCACC ATTAGAATAT TAGATTTTAG TGTTTAAATA

t_MSE SM10 401 GTGCATTTTA CTCTTGCACC ATTAGAATAT TAGATTTTAG TGTTTAAATA

**SM5**

**SM6**

t_MSE 443 AACTAATTTG AGAATTCAAG ATCATAATAT GCATACTAAT TAGACAGTCT

t_MSE SM1 451 AACTAATTTG AGAATTCAAG ATCATAATAT GCATACTAAT TAGACAGTCT

t_MSE SM2 451 AACTAATTTG AGAATTCAAG ATCATAATAT GCATACTAAT TAGACAGTCT

t_MSE SM3 451 AACTAATTTG AGAATTCAAG ATCATAATAT GCATACTAAT TAGACAGTCT

t_MSE SM4 451 AACTAATTTG AGAATTCAAG ATCATAATAT GCATACTAAT TAGACAGTCT

t_MSE SM5 451 cAaTcAgTTG AGAATTCAAG ATCATAATAT GCATACTAAT TAGACAGTCT

t_MSE SM6 451 cAaTcAgTgG cGcAgTaAcG cTaAgAcTcT tCcTcCgAcT gAtAaAtTaT

t_MSE SM7 451 AACTAATTTG AGAATTCAAG ATCATAATAT GCATACTAAT TAGACAGTCT

t_MSE SM8 451 AACTAATTTG AGAATTCAAG ATCATAATAT GCATACTAAT TAGACAGTCT

t_MSE SM9 451 AACTAATTTG AGAATTCAAG ATCATAATAT GCATACTAAT TAGACAGTCT

t_MSE SM10 451 AACTAATTTG AGAATTCAAG ATCATAATAT GCATACTAAT TAGACAGTCT

**SM6**

t_MSE 493 CTTTTTTTTA TTACTTCAAC TATTCAAATT TGCGTTTTTA TTACATTATA

t_MSE SM1 501 CTTTTTTTTA TTACTTCAAC TATTCAAATT TGCGTTTTTA TTACATTATA

t_MSE SM2 501 CTTTTTTTTA TTACTTCAAC TATTCAAATT TGCGTTTTTA TTACATTATA

t_MSE SM3 501 CTTTTTTTTA TTACTTCAAC TATTCAAATT TGCGTTTTTA TTACATTATA

t_MSE SM4 501 CTTTTTTTTA TTACTTCAAC TATTCAAATT TGCGTTTTTA TTACATTATA

t_MSE SM5 501 CTTTTTTTTA TTACTTCAAC TATTCAAATT TGCGTTTTTA TTACATTATA

t_MSE SM6 501 aTgTgTgTgA gTcCgTaAcC gAgTaAcAgT gGCGTTTTTA TTACATTATA

t_MSE SM7 501 CTTTTTTTTA TTACTTCAAC gAgTaAcAgT gGaGgTgTgA gTcCcTgAgA

t_MSE SM8 501 CTTTTTTTTA TTACTTCAAC TATTCAAATT TGCGTTTTTA TTACATTATA

t_MSE SM9 501 CTTTTTTTTA TTACTTCAAC TATTCAAATT TGCGTTTTTA TTACATTATA

t_MSE SM10 501 CTTTTTTTTA TTACTTCAAC TATTCAAATT TGCGTTTTTA TTACATTATA

t_MSE 543 ATTTTCAAGT GGTCTTGGTG CTTTCCAACT GCTAGGATTG AGTTGAAACA

t_MSE SM1 551 ATTTTCAAGT GGTCTTGGTG CTTTCCAACT GCTAGGATTG AGTTGAAACA

t_MSE SM2 551 ATTTTCAAGT GGTCTTGGTG CTTTCCAACT GCTAGGATTG AGTTGAAACA

t_MSE SM3 551 ATTTTCAAGT GGTCTTGGTG CTTTCCAACT GCTAGGATTG AGTTGAAACA

t_MSE SM4 551 ATTTTCAAGT GGTCTTGGTG CTTTCCAACT GCTAGGATTG AGTTGAAACA

t_MSE SM5 551 ATTTTCAAGT GGTCTTGGTG CTTTCCAACT GCTAGGATTG AGTTGAAACA

t_MSE SM6 551 ATTTTCAAGT GGTCTTGGTG CTTTCCAACT GCTAGGATTG AGTTGAAACA

t_MSE SM7 551 cTgTgCcAtT tGgCgTtGgG aTgTaCcAaT tCgAtGcTgG cGgTtAcAcA

t_MSE SM8 551 ATTTTCAAGT GGTCTTGGTG CTTTCCAACT GCTAGGATTG AGTTtAcAcA

t_MSE SM9 551 ATTTTCAAGT GGTCTTGGTG CTTTCCAACT GCTAGGATTG AGTTGAAACA

t_MSE SM10 551 ATTTTCAAGT GGTCTTGGTG CTTTCCAACT GCTAGGATTG AGTTGAAACA

t_MSE 593 AATAAATAAA TAAACAAATA AGTTAACCTT TTGTTTATTA CTTCTAACAA

t_MSE SM1 601 AATAAATAAA TAAACAAATA AGTTAACCTT TTGTTTATTA CTTCTAACAA

t_MSE SM2 601 AATAAATAAA TAAACAAATA AGTTAACCTT TTGTTTATTA CTTCTAACAA

t_MSE SM3 601 AATAAATAAA TAAACAAATA AGTTAACCTT TTGTTTATTA CTTCTAACAA

t_MSE SM4 601 AATAAATAAA TAAACAAATA AGTTAACCTT TTGTTTATTA CTTCTAACAA

t_MSE SM5 601 AATAAATAAA TAAACAAATA AGTTAACCTT TTGTTTATTA CTTCTAACAA

t_MSE SM6 601 AATAAATAAA TAAACAAATA AGTTAACCTT TTGTTTATTA CTTCTAACAA

t_MSE SM7 601 cAgAcATAAA TAAACAAATA AGTTAACCTT TTGTTTATTA CTTCTAACAA

t_MSE SM8 601 cAgAcAgAcA gAcAaAcAgA cGgTcAaCgT gTtTgTcTgA aTgCgAcCcA

t_MSE SM9 601 AATAAATAAA TAAACAAATA AGTTAACCTT TTGTTTATTA CTTCTAACAA

t_MSE SM10 601 AATAAATAAA TAAACAAATA AGTTAACCTT TTGTTTATTA CTTCTAACAA

t_MSE 643 CTTGATTCCT AGAAATTGAA TAACATTTCT TTAAGTGTTT ACAAACATTT

t_MSE SM1 651 CTTGATTCCT AGAAATTGAA TAACATTTCT TTAAGTGTTT ACAAACATTT

t_MSE SM2 651 CTTGATTCCT AGAAATTGAA TAACATTTCT TTAAGTGTTT ACAAACATTT

t_MSE SM3 651 CTTGATTCCT AGAAATTGAA TAACATTTCT TTAAGTGTTT ACAAACATTT

t_MSE SM4 651 CTTGATTCCT AGAAATTGAA TAACATTTCT TTAAGTGTTT ACAAACATTT

t_MSE SM5 651 CTTGATTCCT AGAAATTGAA TAACATTTCT TTAAGTGTTT ACAAACATTT

t_MSE SM6 651 CTTGATTCCT AGAAATTGAA TAACATTTCT TTAAGTGTTT ACAAACATTT

t_MSE SM7 651 CTTGATTCCT AGAAATTGAA TAACATTTCT TTAAGTGTTT ACAAACATTT

t_MSE SM8 651 aTgGcTgCaT cGcAcTgGcA gAcCcTgTaT TTAAGTGTTT ACAAACATTT

t_MSE SM9 651 CTTGATTCCT AGAAATTGcA gAcCcTgTaT gTcAtTtTgT cCcAcCcTgT

t_MSE SM10 651 CTTGATTCCT AGAAATTGAA TAACATTTCT TTAAGTGTTT ACAAACATTT

t_MSE 693 ATTTATTTAT CGCTTAAATC TGAAAACTGA CTATCGACTT ACGAAGCTAG

t_MSE SM1 701 ATTTATTTAT CGCTTAAATC TGAAAACTGA CTATCGACTT ACGAAGCTAG

t_MSE SM2 701 ATTTATTTAT CGCTTAAATC TGAAAACTGA CTATCGACTT ACGAAGCTAG

t_MSE SM3 701 ATTTATTTAT CGCTTAAATC TGAAAACTGA CTATCGACTT ACGAAGCTAG

t_MSE SM4 701 ATTTATTTAT CGCTTAAATC TGAAAACTGA CTATCGACTT ACGAAGCTAG

t_MSE SM5 701 ATTTATTTAT CGCTTAAATC TGAAAACTGA CTATCGACTT ACGAAGCTAG

t_MSE SM6 701 ATTTATTTAT CGCTTAAATC TGAAAACTGA CTATCGACTT ACGAAGCTAG

t_MSE SM7 701 ATTTATTTAT CGCTTAAATC TGAAAACTGA CTATCGACTT ACGAAGCTAG

t_MSE SM8 701 ATTTATTTAT CGCTTAAATC TGAAAACTGA CTATCGACTT ACGAAGCTAG

t_MSE SM9 701 cTgTcTgTcT aGaTgAcAgC gGcAcAaTtA aTcTaGcCgT cCtAcGaTcG

t_MSE SM10 701 ATTTATTTAT CGCTTAAATC TGAAAACTGA CTATCGACTT ACtAcGaTcG

t_MSE 743 GAAAAAAAAA GTGCAATAAA TAGATCTTAG ATTAGACGGG GTGTTGGAAC

t_MSE SM1 751 GAAAAAAAAA GTGCAATAAA TAGATCTTAG ATTAGACGGG GTGTTGGAAC

t_MSE SM2 751 GAAAAAAAAA GTGCAATAAA TAGATCTTAG ATTAGACGGG GTGTTGGAAC

t_MSE SM3 751 GAAAAAAAAA GTGCAATAAA TAGATCTTAG ATTAGACGGG GTGTTGGAAC

t_MSE SM4 751 GAAAAAAAAA GTGCAATAAA TAGATCTTAG ATTAGACGGG GTGTTGGAAC

t_MSE SM5 751 GAAAAAAAAA GTGCAATAAA TAGATCTTAG ATTAGACGGG GTGTTGGAAC

t_MSE SM6 751 GAAAAAAAAA GTGCAATAAA TAGATCTTAG ATTAGACGGG GTGTTGGAAC

t_MSE SM7 751 GAAAAAAAAA GTGCAATAAA TAGATCTTAG ATTAGACGGG GTGTTGGAAC

t_MSE SM8 751 GAAAAAAAAA GTGCAATAAA TAGATCTTAG ATTAGACGGG GTGTTGGAAC

t_MSE SM9 751 tAcAAAAAAA GTGCAATAAA TAGATCTTAG ATTAGACGGG GTGTTGGAAC

t_MSE SM10 751 tAcAcAcAcA tTtCcAgAcA gAtAgCgTcG cTgAtAaGtG tTtTgGtAcC

t_MSE 793 GCCTACAGAT AGGGCCCACC ACTGTACTGG TGATAGCTAC TGGATCCATA

t_MSE SM1 801 GCCTACAGAT AGGGCCCACC ACTGTACTGG TGATAGCTAC TGGATCCATA

t_MSE SM2 801 GCCTACAGAT AGGGCCCACC ACTGTACTGG TGATAGCTAC TGGATCCATA

t_MSE SM3 801 GCCTACAGAT AGGGCCCACC ACTGTACTGG TGATAGCTAC TGGATCCATA

t_MSE SM4 801 GCCTACAGAT AGGGCCCACC ACTGTACTGG TGATAGCTAC TGGATCCATA

t_MSE SM5 801 GCCTACAGAT AGGGCCCACC ACTGTACTGG TGATAGCTAC TGGATCCATA

t_MSE SM6 801 GCCTACAGAT AGGGCCCACC ACTGTACTGG TGATAGCTAC TGGATCCATA

t_MSE SM7 801 GCCTACAGAT AGGGCCCACC ACTGTACTGG TGATAGCTAC TGGATCCATA

t_MSE SM8 801 GCCTACAGAT AGGGCCCACC ACTGTACTGG TGATAGCTAC TGGATCCATA

t_MSE SM9 801 GCCTACAGAT AGGGCCCACC ACTGTACTGG TGATAGCTAC TGGATCCATA

t_MSE SM10 801 tCCTACAGAT AGGGCCCACC ACTGTACTGG TGATAGCTAC TGGATCCATA

**SbfI**

t_MSE 843 GCCCTGAACA TGACCCACGT TGTAGCCTGC AGG

t_MSE SM1 851 GCCCTGAACA TGACCCACGT TGTAGCCTGC AGG

t_MSE SM2 851 GCCCTGAACA TGACCCACGT TGTAGCCTGC AGG

t_MSE SM3 851 GCCCTGAACA TGACCCACGT TGTAGCCTGC AGG

t_MSE SM4 851 GCCCTGAACA TGACCCACGT TGTAGCCTGC AGG

t_MSE SM5 851 GCCCTGAACA TGACCCACGT TGTAGCCTGC AGG

t_MSE SM6 851 GCCCTGAACA TGACCCACGT TGTAGCCTGC AGG

t_MSE SM7 851 GCCCTGAACA TGACCCACGT TGTAGCCTGC AGG

t_MSE SM8 851 GCCCTGAACA TGACCCACGT TGTAGCCTGC AGG

t_MSE SM9 851 GCCCTGAACA TGACCCACGT TGTAGCCTGC AGG

t_MSE SM10 851 GCCCTGAACA TGACCCACGT TGTAGCCTGC AGG
