# Supplementary material for: The Evolutionary Origination and Diversification of a Dimorphic Gene Regulatory Network through Parallel Innovations in cis and trans
Source: PLoS Genet. 2015 Apr 2;11(4):e1005136. doi: 10.1371/journal.pgen.1005136 (PMC4383587; doi:10.1371/journal.pgen.1005136)
Supplement: S9 Fig — Blue background indicates the AscI (GGCGCGCC) and SbfI (CCTGCAGG) restriction enzymes sites that were added for cloning into the reporter transgene vector. Gray background and black letters indicates sequences that comprise a scanning mutation of non-complementary transversions and for which there was not a resulting alteration in the male abdomen regulatory activity. Red background and black letters indicates sequences that comprise a scanning mutation of non-complementary transversions and for which the mutant CRE had a reduced regulatory activity in the male abdomen. The region with a green background and white letters indicates the region where the SM5 mutation was originally made. The lower case nucleotide letters indicate the non-complementary transversions. (DOC) [file pgen.1005136.s009.doc]

**AscI**

t_MSE2 1 GGCGCGCCTG AAATAATAAT AAATAATCAG AATGTAAATA TATTATACGT

t_MSE2SM5.1i 1 GGCGCGCCTG AAATAATAAT AAATAATCAG AATGTAAATA TATTATACGT

t_MSE2SM5.2i 1 GGCGCGCCTG AAATAATAAT AAATAATCAG AATGTAAATA TATTATACGT

t_MSE2SM5.3i 1 GGCGCGCCTG AAATAATAAT AAATAATCAG AATGTAAATA TATTATACGT

t_MSE2SM5.4i 1 GGCGCGCCTG AAATAATAAT AAATAATCAG AATGTAAATA TATTATACGT

t_MSE2SM6.1 1 GGCGCGCCTG AAATAATAAT AAATAATCAG AATGTAAATA TATTATACGT

t_MSE2SM6.2 1 GGCGCGCCTG AAATAATAAT AAATAATCAG AATGTAAATA TATTATACGT

t_MSE2SM6.3 1 GGCGCGCCTG AAATAATAAT AAATAATCAG AATGTAAATA TATTATACGT

t_MSE2SM6.4 1 GGCGCGCCTG AAATAATAAT AAATAATCAG AATGTAAATA TATTATACGT

t_MSE2SM6.5 1 GGCGCGCCTG AAATAATAAT AAATAATCAG AATGTAAATA TATTATACGT

t_MSE2SM6.6 1 GGCGCGCCTG AAATAATAAT AAATAATCAG AATGTAAATA TATTATACGT

t_MSE2SM6 1 GGCGCGCCTG AAATAATAAT AAATAATCAG AATGTAAATA TATTATACGT

t_MSE2 51 TTTATAGATA GAATCAAGAC TTAGGATAAT TGCACTAAGT AGTATACTTA

t_MSE2SM5.1i 51 TTTATAGATA GAATCAAGAC TTAGGATAAT TGCACTAAGT AGTATACTTA

t_MSE2SM5.2i 51 TTTATAGATA GAATCAAGAC TTAGGATAAT TGCACTAAGT AGTATACTTA

t_MSE2SM5.3i 51 TTTATAGATA GAATCAAGAC TTAGGATAAT TGCACTAAGT AGTATACTTA

t_MSE2SM5.4i 51 TTTATAGATA GAATCAAGAC TTAGGATAAT TGCACTAAGT AGTATACTTA

t_MSE2SM6.1 51 TTTATAGATA GAATCAAGAC TTAGGATAAT TGCACTAAGT AGTATACTTA

t_MSE2SM6.2 51 TTTATAGATA GAATCAAGAC TTAGGATAAT TGCACTAAGT AGTATACTTA

t_MSE2SM6.3 51 TTTATAGATA GAATCAAGAC TTAGGATAAT TGCACTAAGT AGTATACTTA

t_MSE2SM6.4 51 TTTATAGATA GAATCAAGAC TTAGGATAAT TGCACTAAGT AGTATACTTA

t_MSE2SM6.5 51 TTTATAGATA GAATCAAGAC TTAGGATAAT TGCACTAAGT AGTATACTTA

t_MSE2SM6.6 51 TTTATAGATA GAATCAAGAC TTAGGATAAT TGCACTAAGT AGTATACTTA

t_MSE2SM6 51 TTTATAGATA GAATCAAGAC TTAGGATAAT TGCACTAAGT AGTATACTTA

t_MSE2 101 AATTCCCATT GCCAAGTGAA CCGGTTGGTA TCCAAAGTTG AAGTCA**ATAA**

t_MSE2SM5.1i 101 AATTCCCATT GCCAAGTGAA CCGGTTGGTA TaCcAcGgTt AcGgCcAgAc

t_MSE2SM5.2i 101 AATTCCCATT GCCAAGTGAA CCGGTTGGTA TCCAAAGTTG AAGTCAcTcA

t_MSE2SM5.3i 101 AATTCCCATT GCCAAGTGAA CCGGTTGGTA TCCAAAGTTG AAGTCAATAA

t_MSE2SM5.4i 101 AATTCCCATT GCCAAGTGAA CCGGTTGGTA TCCAAAGTTG AAGTCAATAA

t_MSE2SM6.1 101 AATTCCCATT GCCAAGTGAA CCGGTTGGTA TCCAAAGTTG AAGTCAATAA

t_MSE2SM6.2 101 AATTCCCATT GCCAAGTGAA CCGGTTGGTA TCCAAAGTTG AAGTCAATAA

t_MSE2SM6.3 101 AATTCCCATT GCCAAGTGAA CCGGTTGGTA TCCAAAGTTG AAGTCAATAA

t_MSE2SM6.4 101 AATTCCCATT GCCAAGTGAA CCGGTTGGTA TCCAAAGTTG AAGTCAATAA

t_MSE2SM6.5 101 AATTCCCATT GCCAAGTGAA CCGGTTGGTA TCCAAAGTTG AAGTCAATAA

t_MSE2SM6.6 101 AATTCCCATT GCCAAGTGAA CCGGTTGGTA TCCAAAGTTG AAGTCAATAA

t_MSE2SM6 101 AATTCCCATT GCCAAGTGAA CCGGTTGGTA TCCAAAGTTG AAGTCAATAA

SM5

t_MSE2 151 CAAAAATGAG TGCATTTTAC TCTTGCACCA TTAGAATATT AGATTTTAGT

t_MSE2SM5.1i 151 CAAAAATGAG TGCATTTTAC TCTTGCACCA TTAGAATATT AGATTTTAGT

t_MSE2SM5.2i 151 aAcAcAgGcG gGaAgTTTAC TCTTGCACCA TTAGAATATT AGATTTTAGT

t_MSE2SM5.3i 151 CAAAAATGAG TtCcTgTgAa TaTgGaAaCa TTAGAATATT AGATTTTAGT

t_MSE2SM5.4i 151 CAAAAATGAG TGCATTTTAC TCTTGCcCaA gTcGcAgAgT cGcTgTTAGT

t_MSE2SM6.1 151 CAAAAATGAG TGCATTTTAC TCTTGCACCA TTAGAATATT cGcTgTgAtT

t_MSE2SM6.2 151 CAAAAATGAG TGCATTTTAC TCTTGCACCA TTAGAATATT AGATTTTAGT

t_MSE2SM6.3 151 CAAAAATGAG TGCATTTTAC TCTTGCACCA TTAGAATATT AGATTTTAGT

t_MSE2SM6.4 151 CAAAAATGAG TGCATTTTAC TCTTGCACCA TTAGAATATT AGATTTTAGT

t_MSE2SM6.5 151 CAAAAATGAG TGCATTTTAC TCTTGCACCA TTAGAATATT AGATTTTAGT

t_MSE2SM6.6 151 CAAAAATGAG TGCATTTTAC TCTTGCACCA TTAGAATATT AGATTTTAGT

t_MSE2SM6 151 CAAAAATGAG TGCATTTTAC TCTTGCACCA TTAGAATATT AGATTTTAGT

**M1** **M2** **M3 M4**

t_MSE2 201 GTTTAAATAA ACTAATTTGA GAATTCAAGA TCATAATATG CATACTAATT

t_MSE2SM5.1i 201 GTTTAAATAA ACTAATTTGA GAATTCAAGA TCATAATATG CATACTAATT

t_MSE2SM5.2i 201 GTTTAAATAA ACTAATTTGA GAATTCAAGA TCATAATATG CATACTAATT

t_MSE2SM5.3i 201 GTTTAAATAA ACTAATTTGA GAATTCAAGA TCATAATATG CATACTAATT

t_MSE2SM5.4i 201 GTTTAAATAA ACTAATTTGA GAATTCAAGA TCATAATATG CATACTAATT

t_MSE2SM6.1 201 tTgTcAcTcA ACTAATTTGA GAATTCAAGA TCATAATATG CATACTAATT

t_MSE2SM6.2 201 GTTTAcAgAc AaTcAgTgGc GcAgTCAAGA TCATAATATG CATACTAATT

t_MSE2SM6.3 201 GTTTAAATAA ACTAATTTGA tAcTgCcAtA gCcTcAgAgG CATACTAATT

t_MSE2SM6.4 201 GTTTAAATAA ACTAATTTGA GAATTCAAGA TCATcAgAgG aAgAaTcAgT

t_MSE2SM6.5 201 GTTTAAATAA ACTAATTTGA GAATTCAAGA TCATAATATG CATACTAATg

t_MSE2SM6.6 201 GTTTAAATAA ACTAATTTGA GAATTCAAGA TCATAATATG CATACTAATT

t_MSE2SM6 201 GTTTAcAgAc AaTcAgTgGc GcAgTaAcGc TaAgAcTcTt CcTcCgAcTg

**M5**

t_MSE2 251 AGACAGTCTC TTTTTTTTAT TACTTCAACT ATTCAAATTT GCGTTTTTAT

t_MSE2SM5.1i 251 AGACAGTCTC TTTTTTTTAT TACTTCAACT ATTCAAATTT GCGTTTTTAT

t_MSE2SM5.2i 251 AGACAGTCTC TTTTTTTTAT TACTTCAACT ATTCAAATTT GCGTTTTTAT

t_MSE2SM5.3i 251 AGACAGTCTC TTTTTTTTAT TACTTCAACT ATTCAAATTT GCGTTTTTAT

t_MSE2SM5.4i 251 AGACAGTCTC TTTTTTTTAT TACTTCAACT ATTCAAATTT GCGTTTTTAT

t_MSE2SM6.1 251 AGACAGTCTC TTTTTTTTAT TACTTCAACT ATTCAAATTT GCGTTTTTAT

t_MSE2SM6.2 251 AGACAGTCTC TTTTTTTTAT TACTTCAACT ATTCAAATTT GCGTTTTTAT

t_MSE2SM6.3 251 AGACAGTCTC TTTTTTTTAT TACTTCAACT ATTCAAATTT GCGTTTTTAT

t_MSE2SM6.4 251 cGcCAGTCTC TTTTTTTTAT TACTTCAACT ATTCAAATTT GCGTTTTTAT

t_MSE2SM6.5 251 AtAaAtTaTa TgTgTgTgAT TACTTCAACT ATTCAAATTT GCGTTTTTAT

t_MSE2SM6.6 251 AGACAGTCTC TTTTTgTgAg TcCgTaAcCg AgTaAcAgTg GCGTTTTTAT

t_MSE2SM6 251 AtAaAtTaTa TgTgTgTgAg TcCgTaAcCg AgTaAcAgTg GCGTTTTTAT

t_MSE2 301 TACATTATAA TTTTCAAGTG GTCTTGGTGC TTTCCAACTG CTAGGATTGA

t_MSE2SM5.1i 301 TACATTATAA TTTTCAAGTG GTCTTGGTGC TTTCCAACTG CTAGGATTGA

t_MSE2SM5.2i 301 TACATTATAA TTTTCAAGTG GTCTTGGTGC TTTCCAACTG CTAGGATTGA

t_MSE2SM5.3i 301 TACATTATAA TTTTCAAGTG GTCTTGGTGC TTTCCAACTG CTAGGATTGA

t_MSE2SM5.4i 301 TACATTATAA TTTTCAAGTG GTCTTGGTGC TTTCCAACTG CTAGGATTGA

t_MSE2SM6.1 301 TACATTATAA TTTTCAAGTG GTCTTGGTGC TTTCCAACTG CTAGGATTGA

t_MSE2SM6.2 301 TACATTATAA TTTTCAAGTG GTCTTGGTGC TTTCCAACTG CTAGGATTGA

t_MSE2SM6.3 301 TACATTATAA TTTTCAAGTG GTCTTGGTGC TTTCCAACTG CTAGGATTGA

t_MSE2SM6.4 301 TACATTATAA TTTTCAAGTG GTCTTGGTGC TTTCCAACTG CTAGGATTGA

t_MSE2SM6.5 301 TACATTATAA TTTTCAAGTG GTCTTGGTGC TTTCCAACTG CTAGGATTGA

t_MSE2SM6.6 301 TACATTATAA TTTTCAAGTG GTCTTGGTGC TTTCCAACTG CTAGGATTGA

t_MSE2SM6 301 TACATTATAA TTTTCAAGTG GTCTTGGTGC TTTCCAACTG CTAGGATTGA

**SbfI**

t_MSE2 351 GTTGAAACAC CTGCAGG

t_MSE2SM5.1i 351 GTTGAAACAC CTGCAGG

t_MSE2SM5.2i 351 GTTGAAACAC CTGCAGG

t_MSE2SM5.3i 351 GTTGAAACAC CTGCAGG

t_MSE2SM5.4i 351 GTTGAAACAC CTGCAGG

t_MSE2SM6.1 351 GTTGAAACAC CTGCAGG

t_MSE2SM6.2 351 GTTGAAACAC CTGCAGG

t_MSE2SM6.3 351 GTTGAAACAC CTGCAGG

t_MSE2SM6.4 351 GTTGAAACAC CTGCAGG

t_MSE2SM6.5 351 GTTGAAACAC CTGCAGG

t_MSE2SM6.6 351 GTTGAAACAC CTGCAGG

t_MSE2SM6 351 GTTGAAACAC CTGCAGG
