# Supplementary material for: The Evolutionary Origination and Diversification of a Dimorphic Gene Regulatory Network through Parallel Innovations in cis and trans
Source: PLoS Genet. 2015 Apr 2;11(4):e1005136. doi: 10.1371/journal.pgen.1005136 (PMC4383587; doi:10.1371/journal.pgen.1005136)
Supplement: S11 Fig — Blue background indicates the AscI (GGCGCGCC) and SbfI (CCTGCAGG) restriction enzymes sites that were added for cloning into the reporter transgene vector. Black background indicates Hox site sequences that were mutated, with the substituted nucleotides shown in lowercase red letters. Teal background indicates the Hth site that was mutated, with the substituted nucleotides shown in white letters. (DOC) [file pgen.1005136.s011.doc]

t_MSE2 1 GGCGCGCCTG AAATAATAAT AAATAATCAG AATGTAAATA TATTATACGT

t_MSE2 TTAT KO 1 GGCGCGCCTG AAATAATAAT AAATAATCAG AATGTAAATA TATTATACGT

t_MSE2 TTAT+TAAT KO 1 GGCGCGCCTG AAATAATAAT AAATAATCAG AATGTAAATA TATTATACGT

t_MSE2 Hth KO 1 GGCGCGCCTG AAATAATAAT AAATAATCAG AATGTAAATA TATTATACGT

t_MSE2 51 TTTATAGATA GAATCAAGAC TTAGGATAAT TGCACTAAGT AGTATACTTA

t_MSE2 TTAT KO 51 TTTATAGATA GAATCAAGAC TTAGGATAAT TGCACTAAGT AGTATACTTA

t_MSE2 TTAT+TAAT KO 51 TTTATAGATA GAATCAAGAC TTAGGATAAT TGCACTAAGT AGTATACTTA

t_MSE2 Hth KO 51 TTTATAGATA GAATCAAGAC TTAGGATAAT TGCACTAAGT AGTATACTTA

t_MSE2 101 AATTCCCATT GCCAAGTGAA CCGGTTGGTA TCCAAAGTTG AAGTCAATAA

t_MSE2 TTAT KO 101 AATTCCCATT GCCAAGTGAA CCGGTTGGTA TCCAAAGTTG AAGTCAATAA

t_MSE2 TTAT+TAAT KO 101 AATTCCCATT GCCAAGTGAA CCGGTTGGTA TCCAAAGTTG AAGTCAATAA

t_MSE2 Hth KO 101 AATTCCCATT GCCAAGTGAA CCGGTTGGTA TCCAAAGTTG AAGTCAATAA

t_MSE2 151 CAAAAATGAG TGCATTTTAC TCTTGCACCA TTAGAATATT AGATTTTAGT

t_MSE2 TTAT KO 151 CAAAAATGAG TGCATTTTAC TCTTGCACCA TTAGAATATT AGATTTTAGT

t_MSE2 TTAT+TAAT KO 151 CAAAAATGAG TGCATTTTAC TCTTGCACCA TTAGAATATT AGATTTTAGT

t_MSE2 Hth KO 151 CAAAAATGAG TGCATTTTAC TCTTGCACCA TTAGAATATT AGATTTTAGT

t_MSE2 201 GTTTAAATAA ACTAATTTGA GAATTCAAGA TCATAATATG CATACTAATT

t_MSE2 TTAT KO 201 GTTTAAcggc ACTAATTTGA GAATTCAAGA TCcggcTATG CATACTAATT

t_MSE2 TTAT+TAAT KO 201 GTTTAAcggc ACcggcTTGA GAATTCAAGA TCcggcTATG CATACcggcg

t_MSE2 Hth KO 201 GTTTAAATAA ACTAATTTGA GAATTCAAGA TCATAATATG CATACTAATT

t_MSE2 251 AGACAGTCTC TTTTTTTTAT TACTTCAACT ATTCAAATTT GCGTTTTTAT

t_MSE2 TTAT KO 251 AGACAGTCTC TTTTTTcggc TACTTCAACT ATTCAAATTT GCGTTTTTAT

t_MSE2 TTAT+TAAT KO 251 gGACAGTCTC TTTTTTcggc cgCTTCAACT ATTCAAATTT GCGTTTTTAT

t_MSE2 Hth KO 251 CCCCCCTCTC TTTTTTTTAT TACTTCAACT ATTCAAATTT GCGTTTTTAT

t_MSE2 301 TACATTATAA TTTTCAAGTG GTCTTGGTGC TTTCCAACTG CTAGGATTGA

t_MSE2 TTAT KO 301 TACATTATAA TTTTCAAGTG GTCTTGGTGC TTTCCAACTG CTAGGATTGA

t_MSE2 TTAT+TAAT KO 301 TACATTATAA TTTTCAAGTG GTCTTGGTGC TTTCCAACTG CTAGGATTGA

t_MSE2 Hth KO 301 TACATTATAA TTTTCAAGTG GTCTTGGTGC TTTCCAACTG CTAGGATTGA

t_MSE2 351 GTTGAAACAC CTGCAGG

t_MSE2 TTAT KO 351 GTTGAAACAC CTGCAGG

t_MSE2 TTAT+TAAT KO 351 GTTGAAACAC CTGCAGG

t_MSE2 Hth KO 351 GTTGAAACAC CTGCAGG
