# Supplementary material for: The Evolutionary Origination and Diversification of a Dimorphic Gene Regulatory Network through Parallel Innovations in cis and trans
Source: PLoS Genet. 2015 Apr 2;11(4):e1005136. doi: 10.1371/journal.pgen.1005136 (PMC4383587; doi:10.1371/journal.pgen.1005136)
Supplement: S1 Table — (DOCX) [file pgen.1005136.s013.docx]

| **Table S1.** Primers used to create *in situ* probes | | | |
| --- | --- | --- | --- |
| **Species** | **gene** | **Primer F** | **Primer R** |
| *D. melanogaster* | *tan* | GTYAAGGAGGAGCACTTYATGTCCCT | taatacgactcactataggGCACTGATSGTRTTGATGCTGAAGACC |
| *D. melanogaster* | *yellow* | GGATTCCGGCCACTCTGACCTATA | TCCGCTCAAGAAAATTGCGTAAAC |
| *D. auraria* | *tan* | CTGYTGGCCACCAGCAAYGTGGAYG | CATGTGSCGSACATCYTGYTCGCTC |
| *D. auraria* | *yellow* | GGGGAYTGCGCSAACAGYATYACCAC | TGGGRAABAGRTGGGGVCCRCTBG |
| *D. kikkawai* | *tan* | TCTGATGATCGACTCTAGCGTC | taatacgactcactataggATTGTCCGAGTACAGGGACATG |
| *D. kikkawai* | *yellow* | TTACTCCTGGGAGCTGAACAAG | taatacgactcactataggCCACAAAGTCATGGTAGCTGTC |
| *D. malerkotliana* | *tan* | YTCSAGCATCTCCAGGATGCARA | taatacgactcactataggGCTCMGTCTCGCTGGGATTGTC |
| *D. malerkotliana* | *yellow* | CCTACATCAACATGGACCACAG | taatacgactcactatagggagaGAARGTGTTCGGATTGGTGTCC |
| *D. ananassae* | *tan* | CAGGCCAATGAGCTGATGATTG | taatacgactcactataggTGTCCGAGTACAAGGACATCGT |
| *D. ananassae* | *yellow* | CGATCTGAGGAACAATGCCTAC | taatacgactcactataggATATATACGCCTTGGGCACCTC |
| *D. pseudoobscura* | *tan* | TTCGATCGCATTCACCAGGATC | taatacgactcactataggTGTCCGAATAGAGCGACATGGT |
| *D. pseudoobscura* | *yellow* | AGGACAGCTACCACGACTTTGT | taatacgactcactataggGTTCTCATCGATCTTCACGTCG |
| *D. willistoni* | *tan* | GGGTGATAAGCAAGAGTTGTTC | taatacgactcactatagggagaTTACCATATCGAAGCCGACCTC |
| *D. willistoni* | *yellow* | CCGGAATTGATACCATATCCGG | taatacgactcactatagggagaATCGGGGAAGAAGTACGAATGG |

**Notes:**

1) The lowercase letters in the table represent the T7 promoter that was added to the reverse primer for in vitro transcription.

2) Probes that lack these sequences were cloned into a pGEM vector, and the insert was amplified from the vector, upon which the probe synthesis reaction was performed to generate an antisense probe.
