# Supplementary material for: The Evolutionary Origination and Diversification of a Dimorphic Gene Regulatory Network through Parallel Innovations in cis and trans
Source: PLoS Genet. 2015 Apr 2;11(4):e1005136. doi: 10.1371/journal.pgen.1005136 (PMC4383587; doi:10.1371/journal.pgen.1005136)
Supplement: S2 Table — (DOCX) [file pgen.1005136.s014.docx]

| **Table S2.** Primers to clone *D. melanogaster yellow* and *tan* CREs | | | |
| --- | --- | --- | --- |
| **Transgene** | **~Size** | **Primer** | **Sequence** |
| y 5’ 1 (wing/body) | 2600 bb | y -2869 Fwd | ggcgcgccCGACTATTAAATGATTATCGCCCG |
|  |  | y -269 Rvs | cctgcaggGTTTGGTATGATTTTTGGCCTTCATC |
| yBE 1.1 | 1100 bb | BE2 Fwd | ggcgcgccGTAAATACACCATTTCATTACACAAC |
|  |  | BE5 Rvs | cctgcaggTAATACATGACAGTTGTGTTCTGAG |
| yBE 0.9 | 900 bp | BE2 Fwd | ggcgcgccGTAAATACACCATTTCATTACACAAC |
|  |  | BE4 Rvs | cctgcaggTACTATTAAATTGGAACTCGTGCTC |
| yBE 0.6 | 600 bp | BE2.5 Fwd | ggcgcgccCTGTGGGTGCAATGATTTAGAATG |
|  |  | BE3.5 Rvs | cctgcaggGTTATTGGCAGGTGATTTTGAGC |
| t_MSE mel | 868 bp | tan MSE deep F | ggcgcgccCCATGGAAGCCGAGCACCTGGTAGA |
|  |  | tan MSE deep R | cctgcaggCTACAACGTRGGTCATGTNCAGGG |
| t_MSE 1 | 374 bp | tan MSE F | ggcgcgccGCAGGACCCGACCCAGATGGCCGCTCAT |
|  |  | tan_MSE-right-R | cctgcaggAATGGTGCAAGAGTAAAATGCACTCA |
| t_MSE 2 | 350 bp | tan_MSE-mid-F | ggcgcgccTGAAATAATAATAAATAATCAGAAT |
|  |  | tan_MSE-mid-R | cctgcaggTGTTTCAACTCAATCCTAGCAGTTGG |
| t_MSE 3 | 373 bp | tan_MSE-left-F | ggcgcgccTTGAGAATTCAAGATCATAATATGCA |
|  |  | tan MSE R | cctgcaggCCAGTACAGTGGTGGGCCCTATCTGTAG |

**Notes:**

1. ‘ggcgcgcc’ and ‘cctgcagg’ are sequences recognized respectively by the AscI and SbfI restriction endonucleases. These restriction enzyme sites were used to clone PCR amplified sequences into the S3aG EGFP reporter vector.

2. Degenerate positions included in primer sequences utilize the IUPAC nucleic acid code: R (A or G), N (A, C, G, or T), S (C or G), M (C or A), Y (C or T), K (T or G), and W (A or T).
