# Supplementary material for: The Evolutionary Origination and Diversification of a Dimorphic Gene Regulatory Network through Parallel Innovations in cis and trans
Source: PLoS Genet. 2015 Apr 2;11(4):e1005136. doi: 10.1371/journal.pgen.1005136 (PMC4383587; doi:10.1371/journal.pgen.1005136)
Supplement: S3 Table — (DOCX) [file pgen.1005136.s015.docx]

| **Table S3.** Primers used to create reporter transgenes with orthologous *yellow* 5' and *t_MSE* sequences | | | | |
| --- | --- | --- | --- | --- |
| **Species** | **Transgene** | **~Size** | **Primer** | **Sequence** |
| D. willistoni | y wil Left | 3274 bp | ywilLrgF | ggcgcgccGGAAGGGGCCATCAAGGGTGAATAG |
|  |  |  | ywilLftR | cctgcaggTTGGCCACATCACATCTTCGTCTCC |
| D. willistoni | y wil Middle | 3293 bp | ywilMidF | ggcgcgccGGGTTTCATTTCCTTCACGCCATTT |
|  |  |  | ywilMidR | cctgcaggGACCCTGTTACAATTTCGGTTCTC |
| D. willistoni | y wil Right | 3024 bp | ywilRtF2.0 | ggcgcgccCCCGGCTGAGTGCATAAATTAGCC |
|  |  |  | ywilLrgR | cctgcaggGTAGTATCCTCTTCTGTGAACCGTG |
| D. pseudoobscura | y wb pse | 2468 bp | ywingF (pse) | ggcgcgccCGATTATTAATCGATTACCAGTCGA |
|  |  |  | ybodyR(msg.pse) | cctgcaggGTCTTCCATGATTGATTTTCACGCAT |
| D. auraria | y wb aur | 4273 bp | y 5' new F2 | ggcgcgccAGGATTAYCTNAATGTGGGAGACTATG |
|  |  |  | y 5' new R4 | cctgcaggATCCYCTTCTGTGGACCGTGGC |
| D. malerkotliana | y wb mal | 3577 bp | y wing ana group Fwd | ggcgcgccGAGCGGAACTGGAGCTGTCAAGCGGT |
|  |  |  | y 5' new R4 | cctgcaggATCCYCTTCTGTGGACCGTGGC |
| D. kikkawai | y wb kik | 4332 bp | y 5' new F2 | ggcgcgccAGGATTAYCTNAATGTGGGAGACTATG |
|  |  |  | y 5' new R4 | cctgcaggATCCYCTTCTGTGGACCGTGGC |
| D. ananassae | y wb ana | 3493 bp | y wing ana group Fwd | ggcgcgccGAGCGGAACTGGAGCTGTCAAGCGGT |
|  |  |  | ybodyR(msg.pse) | cctgcaggGTCTTCCATGATTGATTTTCACGCAT |
| D. melanogaster | t_MSE mel | 868 bp | tan MSE deep F | ggcgcgccCCATGGAAGCCGAGCACCTGGTAGA |
|  |  |  | tan MSE deep R | cctgcaggCTACAACGTRGGTCATGTNCAGGG |
| D. pseudoobscura | t_MSE pse | 885 bp | pse tan MSE F1 | ggcgcgccACGCAGATGAAAGTGCAGGACG |
|  |  |  | pse tan MSE R1 | cctgcaggTCAGTACAGTGGGCCCTATCTG |
| D. willistoni | t_MSE wil | 668 bp | wil tan MSE F2 | ggcgcgccCATGAAAGCCAAGCAACTGATAG |
|  |  |  | wil tan MSE R1 | cctgcaggTGGCGGTTACCAATACAATGGAC |
| D. auraria | t_MSE aur | 1207 bp | tMSE mon ori F2 | ggcgcgccAGMCGCAGRTGRAACTGCAGGACC |
|  |  |  | tMSE mon ori R2 | cctgcaggTGTGGGCCATGTCCAGGGCTACGG |
| D. kikkawai | t_MSE kik | 1084 bp | D. kik t_MSE Fwd 2 | ggcgcgccGCCTATGGGGAGGAGGATCCGGC |
|  |  |  | D. kik t_MSE Rvs 2 | cctgcaggGCAGGAGAAACAGGCCCCAAGCCCGC |
| D. malerkotliana | t_MSE mal | 1009 bp | D. mal t_MSE F1 | GCTGCTGACGGAGTAGCTCC |
|  |  |  | D. mal t_MSE R1 | GACCTACAGGGTGATCGAGTC |

Notes:

1. ‘ggcgcgcc’ and ‘cctgcagg’ are sequences recognized respectively by the AscI and SbfI restriction endonucleases. These restriction enzyme sites were used to clone PCR amplified sequences into the S3aG reporter vector.

2. Degenerate positions included in primer sequences utilize the IUPAC nucleic acid code: K (T or G), R (A or G), Y (C or T), N (A, C, G, or T), and M (C or A).
