# Supplementary material for: The Evolutionary Origination and Diversification of a Dimorphic Gene Regulatory Network through Parallel Innovations in cis and trans
Source: PLoS Genet. 2015 Apr 2;11(4):e1005136. doi: 10.1371/journal.pgen.1005136 (PMC4383587; doi:10.1371/journal.pgen.1005136)
Supplement: S4 Table — (DOCX) [file pgen.1005136.s016.docx]

| **Table S4.** Oligonucleotides used to make t_MSE gel shift assay binding sites. | | |
| --- | --- | --- |
| **Binding Site** | **Sequence (5' to 3')** | **Name** |
| Motif 3 | GAGAATTCAAGATCATAATATGTATACTAA | Probe 4 Top |
|  | TTAGTATACATATTATGATCTTGAATTCTC | Probe 4 Bottom |
| Motif 3 Mutant | GAGAATTCAAGATCGCCGTATGTATACTAA | Probe 4 Top B.3 KO |
|  | TTAGTATACATACGGCGATCTTGAATTCTC | Probe 4 Bottom B.3 KO |
| Motif 4 | ATGTATACTAATTAGACAGTCTCTTTTTTT | Probe 5 Top |
|  | AAAAAAAGAGACTGTCTAATTAGTATACAT | Probe 5 Bottom |
| Motif 4 Mutant | ATGTATACGCCGCGGACAGTCTCTTTTTTT | Probe 5 Top A.8 KO |
|  | AAAAAAAGAGACTGTCCGCGGCGTATACAT | Probe 5 Bottom A.8 KO |
| Motif 5 | TTTTTATTACTTCAACTATTCAAATTT | Probe 6 Top |
|  | AAATTTGAATAGTTGAAGTAATAAAAA | Probe 6 Bottom |
| Motif 5 Mutant | TTTTTGCCGCTTCAACTATTCAAATTT | Probe 6 Top B.4 v2 KO |
|  | AAATTTGAATAGTTGAAGCGGCAAAAA | Probe 6 Bottom B.4 V2KO |
| Dll-con | AACTGTCCGCGGGAATGATTTATGGTCCCAAAT | DllR-Con  (Top) |
|  | ATTTGGGACCATAAATCATTCCCGCGGACAGTT | DllR-Con  (Bottom) |
| Dll-con-mut | AACTGTCCGCGGGACGGCGTTCGGGTCCCAAAT | DllR-Con-mut (Top) |
|  | ATTTGGGACCCGAACGCCGTCCCGCGGACAGTT | DllR-Con-mut (Bottom) |
